# Haemoparasite infection risk in multi-host avian system: an integrated analysis

**DOI:** 10.1017/S0031182024000994

**Published:** 2024-09

**Authors:** Edyta Podmokła, Anna Dubiec, Bartosz Pluciński, Bartłomiej Zając, Lars Gustafsson

**Affiliations:** 1 Department of Comparative Anatomy, Institute of Zoology and Biomedical Research, Jagiellonian University, Kraków, Poland; 2 Museum and Institute of Zoology, Polish Academy of Sciences, Warszawa, Poland; 3 Department of Plant Physiology and Biochemistry, Faculty of Biochemistry, Biophysics and Biotechnology, Jagiellonian University, Kraków, Poland; 4 Department of Ecology and Genetics, Evolutionary Biology Centre, Uppsala University, Uppsala, Sweden

**Keywords:** blood parasites, *Haemoproteus*, host density, host–parasite, infection risk, NGS, *Plasmodium*, *Trypanosoma*

## Abstract

Avian blood parasites play a crucial role in wildlife health and ecosystem dynamics, exhibiting heterogeneous spatial distribution influenced by various factors. Although factors underlying heterogeneity in infection with blood parasites have been explored in many avian hosts, their importance in the context of host species and the parasite taxon remains poorly understood, particularly in cohabiting host species. Using next-generation sequencing for parasite screening, we investigate the association between *Haemoproteus*, *Plasmodium* and *Trypanosoma* infections in relation to individual parameters, host densities and landscape features in 3 cavity-nesting passerines: great tit (*Parus major*), blue tit (*Cyanistes caeruleus*) and collared flycatcher (*Ficedula albicollis*) in a highly fragmented forest habitat. Overall, *Haemoproteus* infections predominated, followed by *Plasmodium* and *Trypanosoma*, with great tits most and collared flycatchers least parasitized. There were no common patterns across host species in the probability of infection with locally transmitted parasites from each genus. Specifically, in all cases, the effect of particular parameters, if present, was observed only in 1 host species. Body condition influenced *Haemoproteus* and *Plasmodium* infections differently in tits. Host density, whether own species or all pooled, explained *Haemoproteus* infections in great tits and collared flycatchers, and *Plasmodium* in great tits. Landscape metrics, such as moisture index and distance to coast edge and pastures, affected infection probability in specific host–parasite combinations. Relative risk maps revealed infection risk gradients, but spatial variation repeatability over time was low. Our study highlights the complex dynamics of avian blood parasites in multi-host systems, shedding light on host–parasite interactions in natural ecosystems.

## Graphical abstract

**Figure:**
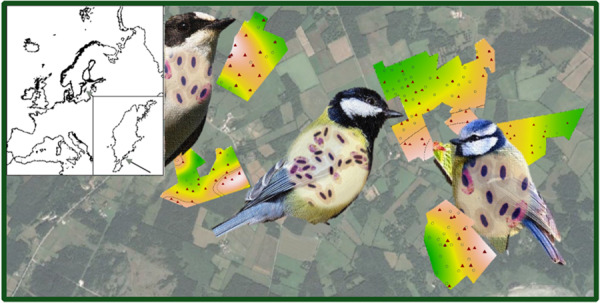

## Introduction

The distribution of organisms in space is a multifaceted phenomenon, influenced by diverse external and internal factors. Climate, landscape and vegetation are some external factors influencing the spatial heterogeneity of organisms. Additionally, population parameters such as density and individual characteristics contribute to their final spatial distribution. Among the many organisms that inhabit natural ecosystems, parasites stand out as a ubiquitous group. They may significantly impact host population dynamics, affecting reproductive success, survival and genetic variability (Schmid-Hempel, 2021). Despite their common occurrence, the distribution of parasites is not uniform and depends on various factors. Identifying these factors is crucial, especially for parasites with significant epidemiological importance, such as those pathogenic to humans or livestock. In the context of wild animal and plant populations, understanding the factors influencing parasite distribution can strongly influence species and habitat protection effectiveness. However, comprehending the relationships between parasites and their environment, particularly for vector-borne parasites, is challenging. The spread of vector-borne parasites involves complex interactions among the parasite, vector, host and the surrounding environment – both biotic and abiotic. It remains unclear whether environmental and climate conditions directly affect parasites (Hance *et al*., 2006; Cardon *et al*., 2011) or do so indirectly *via* hosts or vectors sensitive to climatic conditions and landscape features (Gage *et al*., 2008; Loiseau *et al*., 2012; Ferraguti *et al*., 2016). Although much research has focused on climatic factors, the potential impact of environmental factors (such as vegetation, and the landscape elements) and population parameters has been neglected, proving insufficient to explain spatial distribution patterns of parasites (Pérez-Rodríguez *et al*., 2013; Franklinos *et al*., 2019).

A commonly occurring group of blood parasites in birds includes intra-erythrocytic haemosporidians such as *Plasmodium*, *Haemoproteus* and *Leucocytozoon* (causing avian malaria or similar diseases), along with extracellular trypanosomatids of the genus *Trypanosoma* (Clark *et al*., 2009). These parasites require vectors in their life cycle, with *Plasmodium* transmitted by mosquitoes (Culicidae), *Haemoproteus* by hippoboscid flies (Hippoboscidae) and biting midges (Ceratopogonidae), and *Leucocytozoon* and *Trypanosoma* by Simuliidae and biting midges, among others (Valkiūnas, 2005; Atkinson *et al*., 2008; Svobodová *et al*., 2017).

Although these blood parasites have been detected on all continents except Antarctica (Valkiūnas, 2005), the factors influencing their distribution patterns remain inadequately understood (for a review, see Sehgal, 2015). In the case of avian malaria, landscape features seem to play a significant role (Sehgal, 2015; Ferraguti *et al*., 2018), with prevalence linked to altitude, distance to water bodies, habitat degradation (Knowles *et al*., 2011; Lachish *et al*., 2013; Gonzalez-Quevedo *et al*., 2014; Krama *et al*., 2015; Reinoso-Pérez *et al*., 2016), habitat fragmentation, the amount of edge (Pérez-Rodríguez *et al*., 2018) and land-use and land cover (Ferraguti *et al*., 2023). Factors such as rainfall and vegetation have been identified as good predictors of mosquito abundance and distribution, thus influencing the risk of malaria infections (Roiz *et al*., 2015). For *Trypanosoma*, surface moisture (Sehgal *et al*., 2011) and temperature, precipitation and tree cover have proven to be good predictors of infections, including co-infections with *Leucocytozoon* (Oakgrove *et al*., 2014). Individual traits also explain blood parasite prevalence in birds, with higher prevalence observed in females (McCurdy *et al*., 1998; Valdebenito *et al*., 2023), older individuals (Wood *et al*., 2007) and those in poor condition (Hatchwell *et al*., 2001; Shurulinkov *et al*., 2012). Additionally, co-infections are frequent, and their detection is critical for understanding the complex dynamics of parasitic interactions (Arriero and Møller, 2008; Oakgrove *et al*., 2014; Garcia-Longoria *et al*., 2022). However, polymerase chain reaction (PCR) has shown selective amplification in mixed infections (Zehtindjiev *et al*., 2012) and employing next-generation sequencing (NGS) has revealed much higher parasitic diversity and number of co-infections (Jarvi *et al*., 2013; Galen *et al*., 2020).

Studies addressing spatial heterogeneity of parasite occurrence within single host populations are limited (Wood *et al*., 2007; Knowles *et al*., 2011; Lachish *et al*., 2011; Garroway *et al*., 2013; Kubacka *et al*., 2019), and even rarer are studies encompassing co-occurring multiple host species sampled in the same point of each host annual cycle (Lachish *et al*., 2013). Considering that the impact of parasites on their hosts is dependent on the parasite's taxon (either at the level of genus, species or lineage) and host species (Palinauskas *et al*., 2008; Lachish *et al*., 2011), research that captures the complex reality of different hosts, parasites and potential factors influencing their spatial distribution is crucial. In this study, we employed NGS to identify blood parasites in 3 forest-dwelling passerine species – 2 resident (the great tit *Parus major* and the blue tit *Cyanistes caeruleus*) and 1 migratory (the collared flycatcher *Ficedula albicollis*) – occupying the same breeding area and the same nesting niche, thus experiencing a similar risk of locally transmitted infections, but with shorter exposure to local vectors in the case of the migratory species. We assessed the respective influences of intrinsic factors (individual parameters) and extrinsic factors (population metrics and landscape metrics including high-resolution satellite remote-sensing data reflecting vegetation cover and moisture levels) as drivers of variations in parasitic infection patterns. Also, we investigated spatial variation in infection risk with parasites representing each genus, and examined whether spatial patterns are repeatable over time. We sampled birds in several plots located in a highly fragmented forest surrounded by agricultural landscape. We hypothesized that an increase in distance from the woodland edge and bird density in the vicinity of the nesting site would elevate the probability of infection. We also anticipated that closer proximity to agricultural fields, where pesticides may negatively impact vectors, and increasing distance to pastures, which serve as food reservoirs for vectors, would negatively affect the probability of infections. Additionally, we expected higher susceptibility to infections in older individuals, females (due to their higher reproductive effort and higher exposure to vectors during early stages of the nesting cycle), as well as individuals in poor condition.

## Methods

### Study area and bird sampling

**Figure 1.**
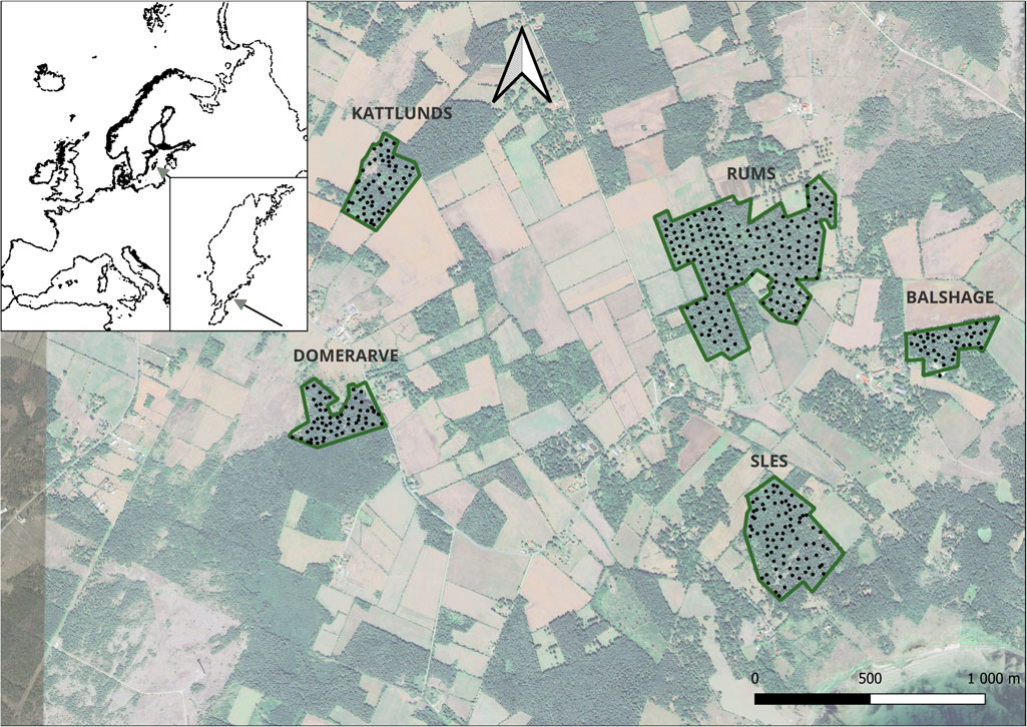
Distribution of research plots (dark green borders) and nest boxes (black circles) in the southern part of the island of Gotland, Sweden.

The study encompassed 2 breeding seasons (2019 and 2021) within wild populations of 3 species of passerine birds: blue tit (BT), great tit (GT) and collared flycatcher (CF). These species inhabited the same breeding area in the southern part of the Swedish island of Gotland (57°03ʹ N, 18°17ʹ E), which is composed of highly fragmented forest surrounded by agricultural landscape. We sampled birds in 5 plots containing in total 424 nestboxes (Fig. 1). The plots underwent regular inspections from the beginning of May to record laying dates, clutch sizes and hatching dates.

We captured 618 adult birds, either with traps mounted inside the nest box or mist-netting in the close vicinity of the nest while feeding the nestlings. Captures occurred when nestlings were approximately 10 days old or older (starting day in 2019: for tits – 12th, for flycatchers – 10th; starting day in 2021: for tits – 10th, for flycatchers – 8th). Bird age was determined based on the colour of the wing coverts (Svensson, 1992), and sex was identified using plumage characteristics (in the case of flycatchers) or the presence/absence of the brood-patch (in the case of tits). Wing length was measured with a wing ruler, tarsus with a calliper and body mass with weighting scales. Blood was sampled through brachial venipuncture and stored in room temperature in 96% ethanol. In total, 609 individuals were sampled.

### Identification and diversity of blood parasites

DNA extraction followed the standard ammonium acetate precipitation method (Bruford *et al*., 1998), and the concentration and quality of extracted DNA were assessed using a NanoDrop spectrophotometer. Working dilutions (25 ng μL^−1^) were prepared for screening samples for the presence of 3 genera of blood parasites: *Haemoproteus*, *Plasmodium* and *Trypanosoma*. Based on the previous studies conducted in these populations (Radwan *et al*., 2012; Podmokła *et al*., 2014; Dubiec *et al*., 2016; Fletcher *et al*., 2019), we anticipated multiple infections, particularly in great tits (Table 1). Thus, we opted for the Illumina NGS platform. This platform allows the detection and sequencing of multiple parasitic lineages in co-infected blood samples. Primers described by Yeo *et al*. (2022) were used for *Haemoproteus* and *Plasmodium*, amplifying a fragment of the mitochondrial cyt b gene (Supplementary Table S1). Those primers were supposed to also amplify a fragment of *Leucocytozoon*, but it was unsuccessful in our case. For *Trypanosoma*, we designed our own primers amplifying a fragment of the small subunit ribosomal RNA (SSU rRNA) (Supplementary Table S1), following a process outlined in the Supplementary Materials (Appendix S1) and all obtained *Trypanosoma* sequences were classified into the 3 groups described by Zídková *et al*. (2012): group A (including *T. bennetti*), group B (including *T. corvi* and *T. culicavium*) and group C (including *T. avium*). To sequence the amplicons on an Illumina platform and successfully demultiplex them in the bioinformatic stage, we implemented a 6 bp unique primer-tag combination in a 1-step PCR reaction (Supplementary Table S1).

**Table 1.** Prevalence and diversity of haemosporidia parasites in the 3 studied bird species based on the previous research conducted on Gotland

Bird species:		Blue tit	Great tit	Collared flycatcher	Collared flycatcher	Collared flycatcher
Paper:		Podmokła *et al.* (2014)	Dubiec *et al*. (2016)	Radwan *et al*. (2012)	Fletcher *et al*. (2019)	Fletcher *et al*. (2019)
Years of study:		2008–2012	2011–2012	2003	2009–2012	2009–2012
		Adults, *N* = 762	Adults, *N* = 324	Adults, *N* = 212	Juveniles, *N* = 700	Adults, *N* = 512
**Parasite Species**	**Lineage**					
***Haemoproteus***		**15.7%**	**46.3%**	**29.7%**	**7.6%**	**17.4%**
*pallidus*	COLL2			8 (3.8%)		13 (2.5%)
*balmorali*	COLL3			2 (0.9%)	9 (1.3%)	13 (2.5%)
	CWT4				1 (0.1%)	
*majoris*	PARUS1	117 (15.4%)	58 (17.9%)			
	PARUS65		1 (0.3%)			
*pallidus*	PFC1			1 (0.5%)	1 (0.1%)	6 (1.2%)
*majoris*	PHSIB1	2 (0.3%)	68 (21%)	52 (24.5%)	41 (5.9%)	56 (10.9%)
*majoris*	WW2	1 (0.1%)	23 (7.1%)		1 (0.1%)	1 (0.2%)
***Plasmodium***		**46.6%**	**78.4%**	**4.7%**	**0.8%**	**4.5%**
	ACCTAC01			1 (0.5%)		1 (0.2%)
	AEMO01					1 (0.2%)
*circumflexum*	BT7	60 (7.9%)	25 (7.7%)			
	COLL6					1 (0.2%)
	COLL7			2 (0.9%)		2 (0.4%)
	GRW09			2 (0.9%)	3 (0.4%)	5 (1%)
	GRW10			2 (0.9%)		
*relictum*	GRW11	2 (0.3%)	1 (0.3%)			1 (0.2%)
	LAMPUR03					4 (0.8%)
	PADOM12			1 (0.5%)		
	PARUS66		3 (0.9%)			
	PARUS67		1 (0.3%)			
	PBPIP1					1 (0.2%)
	PSB1					3 (0.6%)
	RFF1			1 (0.5%)		
	RTSR1			1 (0.5%)		2 (0.4%)
*relictum*	SGS1	16 (2.1%)	37 (11.4%)		1 (0.1%)	1 (0.2%)
*polare*	SW2	2 (0.3%)	56 (17.3%)			
*circumflexum*	TURDUS1	275 (36.1%)	131 (40.4%)		2 (0.3%)	1 (0.2%)
**MI (multiple infections)**		2.8%	26.2%	1.4%	0.3%	1%

#### PCR and library preparation

Fragments of parasite mitochondrial cyt b and SSU rRNA were amplified simultaneously in multiplex PCR. The reaction was performed in 10 μL volume, including 5 μL of Multiplex PCR Master Mix (Qiagen, Hilden, Germany), 2 μL of distilled water, 2 μL of DNA template (in working concentration = 25 ng μL^−1^) and 0.5 μm of F and R primers (mixed in proportion 2:3 for *Haemoproteus*/*Plasmodium* and *Trypanosoma*, respectively). The PCR conditions were as follows: 95°C for 15 min, 35 cycles of 94°C for 30 s, 57°C for 90 s and 72°C for 60 s, followed by 60°C for 30 min. Amplification was performed in duplicates to minimize the impact of PCR amplification on total number of reads per parasite haplotype. The 2 PCR products per individual were pooled together and visualized in agarose gel. A total of 8 libraries were created, containing samples from the current project and another one. In each library, up to 190 products were pooled in equimolar proportions based on the intensity of bands in the gel, pools were gel-purified using Zymoclean™ Gel DNA Recovery Kit (Zymo) and Illumina adaptors were ligated using NEBNext Ultra II FS DNA PCR-free Library Prep Kit (New England Biolabs, Ipswich, MA, USA). Libraries were quantitated with Qubit Fluorometer and sequenced on Illumina MiSeq (v2 500 cycles kit).

#### Bioinformatics

The Illumina raw read files underwent quality checks using FastQC (Andrews, 2010). Trimming and adaptor removal were performed using Trimmomatic (Bolger *et al*., 2014). Subsequent steps, including demultiplexing, dereplication and denoising (with restriction to sequence length ⩾300 bp and sequence count ⩾10), were executed using OBITools3 (Boyer *et al*., 2016). The final FASTA file was BLASTed against a local database created by merging the MalAvi dataset (Bensch *et al*., 2009) (for *Leucocytozoon*, *Haemoproteus* and *Plasmodium*) and the *Trypanosoma* dataset designed for primer development (see Supplementary Appendix S1) based on Zídková *et al*. (2012) using Biopython (Cock *et al*., 2009). Information extraction for the best hits of each unique sequence obtained from every sample, along with their respective counts, was conducted with XML parser's library Beautiful Soup (Richardson, 2023). The final table with extracted data was prepared with the pandas package v. 2.0.3 (The Pandas Development Team, 2020) and the dplyr package v. 1.1.4 (Wickham *et al*., 2023). To ensure the reliability of downstream analysis and prevent potential contamination and misdiagnosis resulting from low coverage, only sequences with counts exceeding 10 per individual were included in the final dataset used for data analysis (refer to Supplementary Table S2, for detailed information on observed sequence counts for each parasite lineage and bird species).

### Landscape and population metrics

We calculated the distance between each nestbox and the borders of: (i) the forest complex, providing a measure known as ‘ForestEdge’; (ii) the nearest seacoast (‘CoastEdge’); (iii) arable fields (‘Field’); (iv) pastures (‘Pasture’) (refer to Supplementary Appendix S2 for methodological details and Table S3 for detailed information on specific distances for each bird species). We also obtained indices of: (i) the local density of all bird species, providing a measure known as ‘AllSpecDens’, and (ii) the local density of individuals of the same species as the analysed species, providing a measure known as ‘OwnSpecDens’.

For the calculation of Normalized Difference Vegetation Index (NDVI), we utilized satellite images from the middle of June (17/06/2019 and 15/06/2021), capturing a representative snapshot of vegetation condition during the active period of adult vectors, and for Normalized Difference Moisture Index (NDMI), we decided to use images from the second half of April (23/04/2019 and 19/04/2021), due to very limited vegetation and quite high soil moisture caused by snowmelt, creating favourable conditions for the development of vector larvae (refer to Supplementary Appendix S2 for methodological details).

### Spatial variation in the relative risk of infection

To examine within-plot spatial variation in infection risk with parasites representing each of the 3 genera (pooled data for the 3 host species), we used maps of the relative risk, which depict the probability of infection relative to distribution of population at-risk (refer to Supplementary Appendix S2 for methodological details). To generate maps, we used fixed-bandwidth kernel smoothing with a shrinkage estimator of a lasso type to shrink a standard kernel estimator of the log-relative risk function towards zero (Hazelton, 2023). To identify areas of the plots with elevated risk of infection (at 0.05 and 0.01 level), *P* value surfaces for shrinkage estimates of the log-relative risk function were calculated using the asymptotic approach (Hazelton and Davies, 2009). The analyses were carried out with an R package sparr (Davies *et al*., 2018).

### Statistical analysis

The data were analysed using the R environment (R Core Team, 2023; R version 4.3.2). To assess the impact of landscape and population metrics, as well as individual variables on infection status (coded as a binary variable: 1 – infected, 0 – uninfected), we employed generalized linear models with a binomial error structure and logit link, fitted using the ‘glm’ function. Separate analyses were conducted for landscape and population metrics, and individual variables. Each analysis, performed independently for each bird species, comprised 3 model sets, with the response variable being infection status based on: (1) *Haemoproteus*, (2) *Plasmodium* and (3) *Trypanosoma*. For *Haemoproteus* and *Plasmodium*, only lineages with known transmission in Europe were considered (based on MalAvi database, refer to Table 2), whereas for *Trypanosoma*, all cases were included since transmission information is limited. Models for *Plasmodium* infections in the collared flycatcher were not analysed due to the too small number of infected individuals (10 out of the 267).

**Table 2.** Blood parasites identified in studied birds species: blue tit, great tit and collared flycatcher

Bird species:		Blue tit	Great tit	Collared flycatcher	Transmission in Europe	used in analysis
Species	Lineage	*N* = 67	*N* = 275	*N* = 267	(Based on MalAvi)	
***Haemoproteus***		**47.8%**	**72.7%**	**36.0%**		
*belopolskyi*	ARW1	1 (1.5%)	8 (2.9%)	4 (1.5%)	Unknown	no^a^
*pallidus*	COLL2			5 (1.9%)	Known	yes
*balmorali*	COLL3	13 (19.4%)	50 (18.2%)	45 (16.9%)	Known	yes
*majoris*	PARUS1	20 (29.9%)	112 (40.7%)		Known	yes
*majoris*	PHSIB1	1 (1.5%)	143 (52%)	46 (17.2%)	Known	yes
	RW2	1 (1.5%)	6 (2.2%)	9 (3.4%)	Known	no^a^
*majoris*	WW2		19 (6.9%)		Known	yes
***Haemoproteus* MI (multiple infections)**		**6.0%**	**39.3%**	**4.1%**		
***Plasmodium***		**26.9%**	**44.4%**	**4%**		
*circumflexum*	BT7	6 (9%)	25 (9.1%)		Known	yes
	COLL7			1 (0.4%)	Unknown	no
	GRW09			1 (0.4%)	Known	yes
*relictum*	GRW11		7 (2.5%)		Known	yes
	LAMPUR03			1 (0.4%)	Unknown	no
	PLOPRI01			2 (0.7%)	Unknown	no
	RTSR1			1 (0.4%)	Unknown	no
*relictum*	SGS1	2 (3%)	19 (6.9%)	1 (0.4%)	Known	yes
*polare*	SW2	1 (1.5%)	61 (22.2%)		Known	yes
*circumflexum*	TURDUS1	11 (16.4%)	33 (12%)		Known	yes
	WW4			3 (1.1%)	Unknown	no
***Plasmodium* MI (multiple infections)**		**3.0%**	**6.9%**	**0.0%**		
***Trypanosoma***		**17.9%**	**9.8%**	**25.1%**		
	group A	9 (13.4%)	17 (6.2%)	15 (5.6%)	Unknown	yes
	group B	1 (1.5%)	1 (0.4%)	40 (15%)	Unknown	yes
	group C	3 (4.5%)	11 (4%)	21 (7.9%)	Unknown	yes
***Trypanosoma* MI (multiple infections)**		**6.0%**	**1.8%**	**6.7%**		

For *Haemoproteus* and *Plasmodium*, information on the transmission was obtained from the MalAvi database (Bensch *et al*., 2009), as of February 2024. A lineage was considered to be transmitted in Europe if it has previously been found in juveniles of a migratory species or adults/juveniles of a resident species.

a Lineages ARW1 and RW2 were excluded from analysis due to doubts about the analysed birds as competent hosts and suspicion of abortive haemosporidians infections.

In the first analysis, we investigated whether infection status could be explained by variables related to the individual: sex, age, body condition and the year of the study. Body condition was estimated using residuals from an ordinary least squares regression of body mass against tarsus length, calculated separately for each sex within each bird species. In the second analysis, we examined whether the infection status could be explained by any of the landscape and population variables. The global model included distance to the woodland edge, coast edge, fields, pastures, NDVI, NDMI and the local density of: (i) all bird species, (ii) the same species. All the numeric landscape and population variables were standardized to a mean of zero and an s.d. of 1 using the ‘mutate’ function from the dplyr package (Wickham *et al*., 2023). In both analyses, the starting model included all variables, without their interactions due to the lack of biological justification for them. All landscape and population variables were checked for correlations (Supplementary Fig. S1) and all global models were diagnosed for collinearity using the ‘vif’ function from the car package (Fox and Weisberg, 2019) and in all cases the variance inflation factor was lower than 3, with maximum value equal to 2.62 (refer to Supplementary Tables S4 and S5). Akaike's information criterion (AIC) was employed to determine the combination of variables that best explained the data (Burnham and Anderson, 2004). Automated model selection was performed using the MuMIn package (Bartoń, 2023) and the ‘dredge’ function. For the blue tit models, we limited the maximum number of variables in a model to 6 to allow approximately 10 data points per estimation (*N*_BT_ = 67) and reduce the risk of over-parametrization (Harrison *et al*., 2018). For model ranking, we used the Akaike information criterion corrected for small sample size (AICc). We also provided quantitative measures of relative support for each model: model likelihood (relative likelihood of the model in the candidate set, given the data), Akaike weight (*ω*AICc; probability that the given model is the best approximating model in the candidate set) and evidence ratio (describing how much more likely the best model is than the given model), as well as cumulative AICc (i.e. sum of AICc values of a given model and all the higher-ranking models) (Symonds and Moussalli, 2011). Subsequently, models with ΔAICc <2 were averaged. For each predictor that appeared in the group of averaged models, an estimate and 95% CI were obtained and used to assess the effect of this predictor on the probability of infection. Specifically, if the 95% confidence intervals did not include zero, we considered the predictor to have an effect.

## Results

### Prevalence and community composition of blood parasites

**Figure 2.**
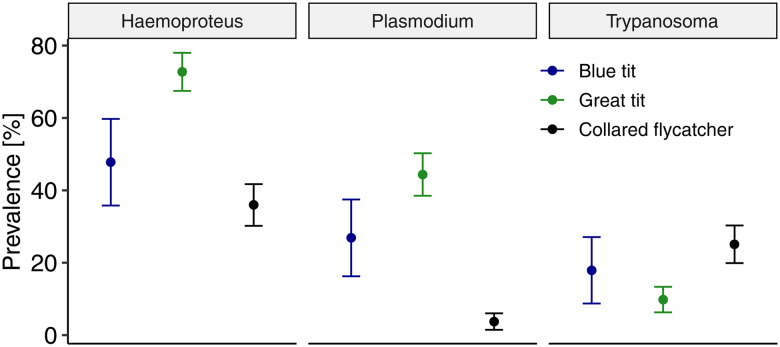
The prevalence of parasites from *Haemoproteus*, *Plasmodium* and *Trypanosoma* genera in 3 avian host species sampled during the breeding season on Gotland, Sweden. Points represent means and 95% confidence intervals.

In total, we detected 7 lineages of *Haemoproteus* and 11 of *Plasmodium* parasites (Table 2). The prevalence of parasites from each genus varied among the study host species (Fig. 2). The prevalence of *Haemoproteus* ranged from 36% in the collared flycatcher to 48% in the blue tit and up to 73% in the great tit. The lineage richness of *Haemoproteus* showed limited variation among bird species, with 5, 6 and 5 lineages in the blue tit, great tit and collared flycatcher, respectively. These represent the minimum numbers of lineages, as some lineages could not be distinguished due to their identical sequences: hPFC1 was indistinguishable from hCOLL2, hCWT4 from hWW2 and hPARUS65 from hPARUS 1. In such cases, we assumed that the lineage marked as more frequent in previous studies (Table 1) was more probable. We found 2 lineages unusual for the studied bird species: hARW1 and hRW2. Although the transmission of one of them (hRW2) was confirmed in Europe (based on the MalAvi database), we decided not to include either of these lineages in further analyses due to uncertainties about the study bird species as competent hosts and suspicion of these infections being of abortive type. Multiple infections with *Haemoproteus* occurred at varying levels, from 4% in the collared flycatcher to 6% in the blue tit and up to 39% in the great tit.

*Plasmodium* prevalence ranged from 4% in the collared flycatcher to 27% in the blue tit and up to 44% in the great tit. The richness of *Plasmodium* lineages varied among hosts, with 4, 5 and 7 detected lineages in the blue tit, great tit and collared flycatcher, respectively. As with *Haemoproteus*, this represents the minimum number of lineages because in the case of pPARUS66, it was indistinguishable from pSW2, and the latter, being more frequent, was considered more probable. Multiple infections with different *Plasmodium* lineages were absent in the collared flycatcher and relatively infrequent in the tits, occurring in 3% of the blue tit and 7% of the great tit.

*Trypanosoma* prevalence was 10% in the great tit, 18% in the blue tit and 25% in the collared flycatcher. Each of the 3 groups of *Trypanosoma* occurred in all studied birds, with group A being the most prevalent in the blue tit and great tit, and group B in the collared flycatcher. Multiple infections with different *Trypanosoma* groups were least common in the great tit (less than 2% of birds) and occurred at similar levels in the blue tit and collared flycatcher (6 and 6.7%, respectively).

### Individual traits

**Figure 3.**
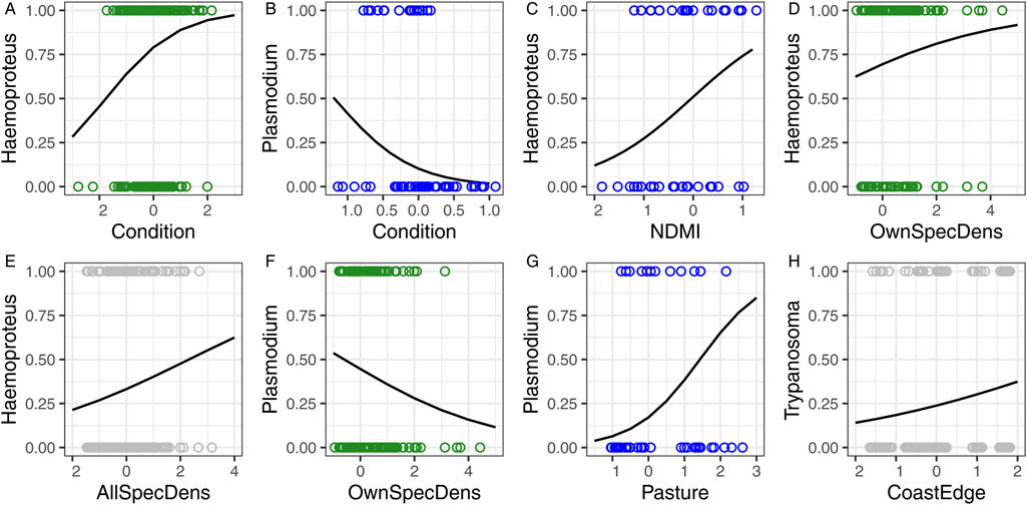
The probability of infection with parasites from genera *Haemoproteus*, *Plasmodium* or *Trypanosoma* (indicated on the *Y* axis) in relation to: (A–B) individual variables, (C–H) landscape and population variables. The lines were fitted based on the estimates from the respective top models in Tables S6 and S7, and the bands show confidence intervals. Circles represent infected (*y* = 1) and uninfected (*y* = 0) individuals, with the circle colour indicating the host species: blue for the blue tit, green for the great tit, grey for the collared flycatcher. Condition – host body condition; NDMI – Normalized Difference Moisture Index; OwnSpecDens – the local density of individuals of the same species; AllSpecDens – the local density of all bird species; CoastEdge – the distance to the nearest seacoast; Pasture – the distance to the nearest pasture.

In all host species × parasite genus combinations, there was uncertainty in selecting the best model. The subset of best models is presented in Table S6 (Supplementary Materials). Among individual traits, only condition was associated with the probability of infection: in great tits, the condition increased with an increasing probability of infection with *Haemoproteus* (Table 3, Fig. 3A), whereas in blue tits, it decreased with the probability of *Plasmodium* infection (Table 3, Fig. 3B). Additionally, infection rates varied between seasons: *Haemoproteus* and *Plasmodium* infection rates fluctuated in both tit species, and *Trypanosoma* infection rates varied in collared flycatchers (Table 3).

**Table 3.** Model-averaged estimates of the effects of individual traits: sex, age, body condition and the year of the study on the status of infection with: (1) *Haemoproteus*; (2) *Plasmodium*; (3) *Trypanosoma*

Term	*Haemoproteus*			*Plasmodium*^a^		*Trypanosoma*		
	Blue tit	Great tit	Collared flycatcher	Blue tit	Great tit	Blue tit	Great tit	Collared flycatcher
**Intercept**	1.15 (0.21; 2.08)	1.29 (0.8; 1.79)	−0.65 (−0.94; −0.35)	−2.19 (−3.58; −0.8)	−1.18 (−1.69; −0.68)	−2.13 (−3.6; −0.66)	−2.23 (−2.87; −1.59)	−1.29 (−1.83; −0.76)
**SEXM**		0.54 (−0.05; 1.13)	−0.21 (−0.73; 0.3)	−0.62 (−1.86; 0.61)	0.36 (−0.21; 0.93)		0.53 (−0.32; 1.39)	0.08 (−0.48; 0.65)
**AGE2+**	0.52 (−0.66; 1.7)	0.41 (−0.23; 1.05)		0.59 (−0.66; 1.83)	0.43 (−0.15; 1.01)	0.76 (−0.59; 2.12)	−0.51 (−1.37; 0.34)	−0.36 (−0.97; 0.25)
**CONDITION**	0.84 (−0.45; 2.13)	**0.75 (0.34; 1.16)**		**−1.86** (**−3.42; −0.3)**	0.32 (−0.06; 0.69)	−1.11 (−2.62; 0.4)	0.44 (−0.11; 0.99)	0.08 (−0.38; 0.54)
**YEAR2021**	**−2.49** (**−3.68; −1.3)**	**−1.77 (−2.39; −1.15)**		**1.62** (**0.21; 3.02)**	**1.99** (**1.41; 2.56)**	1.14 (−0.34; 2.62)	−0.46 (−1.35; 0.44)	**0.57** (**0.01; 1.13)**

Estimates and their 95% confidence intervals (CIs) are shown separately for each bird species: (A) blue tit, (B) great tit; (C) collared flycatcher. The predictors with CIs on the link scale not spanning zero are marked in bold.

a Models for *Plasmodium* infections in the collared flycatcher were not analysed due to the too small number of infected individuals (10 out of the 267).

### Population and landscape metrics

In all host species × parasite genus combinations, there was a high degree of uncertainty in selecting the best model. The subset of best models is presented in Table S7 (Supplementary Materials). Host density, either of the own species or all species pooled, was an important predictor of haemoparasitic infections. Specifically, the density of the own species was positively related to *Haemoproteus* infection rates and negatively related to *Plasmodium* infection rates in the great tit (Table 4, Fig. 3D and F), whereas the density of all species pooled was positively associated with the probability of *Haemoproteus* infection in collared flycatchers (Table 4, Fig. 3E). The moisture index was positively associated with *Haemoproteus* infection rates, but only in blue tits (Table 4, Fig. 3C). Among the 4 parameters describing distance, only distance to pasture and seacoast played a role in explaining haemoparasitic infections. In blue tits, the probability of infection with *Plasmodium* increased with increasing distance from pastures (Table 4, Fig. 3G), and in collared flycatchers, the probability of *Trypanosoma* infection increased with greater distance from the seacoast (Table 4, Fig. 3H).

**Table 4.** Model-averaged estimates of the effects of the landscape and population variables: the local density of all bird species, the local density of the same species, woodland edge, coast edge, NDVI, NDMI, distance to the field, distance to the pasture on the status of infection with: (1) *Haemoproteus*; (2) *Plasmodium*; (3) *Trypanosoma*

Term	*Haemoproteus*			*Plasmodium*^a^		*Trypanosoma*		
	Blue tit	Great tit	Collared flycatcher	Blue tit	Great tit	Blue tit	Great tit	Collared flycatcher
**Intercept**	−0.21 (−3.02; 2.6)	**0.83 (0.57; 1.1)**	**−0.7** (**−0.97; −0.43)**	**−1.46** (**−2.24; −0.68)**	−0.24 (−0.48; 0.01)	**−1.66** (**−2.41; −0.91)**	**−2.25** (**−2.67; −1.84)**	**−1.16** (**−1.45; −0.87)**
**AllSpecDens**	−0.2 (−0.66; 0.27)	−0.15 (−0.45; 0.14)	**0.32 (0.02; 0.63)**	−0.37 (−0.99; 0.25)	−0.07 (−0.35; 0.21)	0.44 (−0.12; 0.99)	−0.09 (−0.5; 0.32)	0.05 (−0.24; 0.34)
**OwnSpecDens**	−2.6 (−12.04; 6.84)	**0.32 (0.01; 0.64)**	−0.17 (−0.49; 0.16)		**−0.36** (**−0.63; −0.08)**		−0.14 (−0.58; 0.3)	−0.12 (−0.41; 0.16)
**ForestEdge**		−0.19 (−0.5; 0.11)	−0.29 (−0.59; 0.01)	−0.85 (−1.74; 0.03)	−0.08 (−0.35; 0.2)		0.12 (−0.25; 0.5)	0.11 (−0.19; 0.41)
**CoastEdge**		−0.06 (−0.35; 0.22)	0.25 (−0.03; 0.53)	0.6 (−0.1; 1.29)	−0.18 (−0.48; 0.12)	0.31 (−0.4; 1.02)	−0.26 (−0.75; 0.24)	**0.33 (0.05; 0.61)**
**NDMI**	**1.02** (**0.25; 1.79)**	0.22 (−0.09; 0.52)		−0.61 (−1.56; 0.34)	−0.27 (−0.56; 0.02)		0.2 (−0.28; 0.68)	−0.09 (−0.34; 0.16)
**NDVI**	−0.19 (−0.83; 0.44)	−0.15 (−0.4; 0.11)	−0.18 (−0.47; 0.11)	0.7 (−0.2; 1.6)	0.04 (−0.2; 0.27)	0.66 (−0.34; 1.66)		−0.1 (−0.4; 0.21)
**Field**	−0.15 (−0.7; 0.4)	−0.12 (−0.38; 0.14)	−0.26 (−0.54; 0.01)	0.48 (−0.17; 1.14)			−0.21 (−0.65; 0.23)	−0.1 (−0.38; 0.18)
**Pasture**	0.28 (−0.19; 0.74)	0.22 (−0.12; 0.56)	0.18 (−0.17; 0.52)	**0.84 (0.02; 1.66)**	0.22 (−0.05; 0.48)	0.41 (−0.35; 1.16)	0.27 (−0.11; 0.64)	

Estimates and their 95% confidence intervals (CIs) are shown separately for each bird species: (A) blue tit, (B) great tit; (C) collared flycatcher.

The predictors were standardized to the mean of zero and standard error of 1, and those with CIs on the link scale not spanning zero are marked in bold.

a Models for *Plasmodium* infections in the collared flycatcher were not analysed due to the too small number of infected individuals (10 out of the 267)

### Relative risk maps

**Figure 4.**
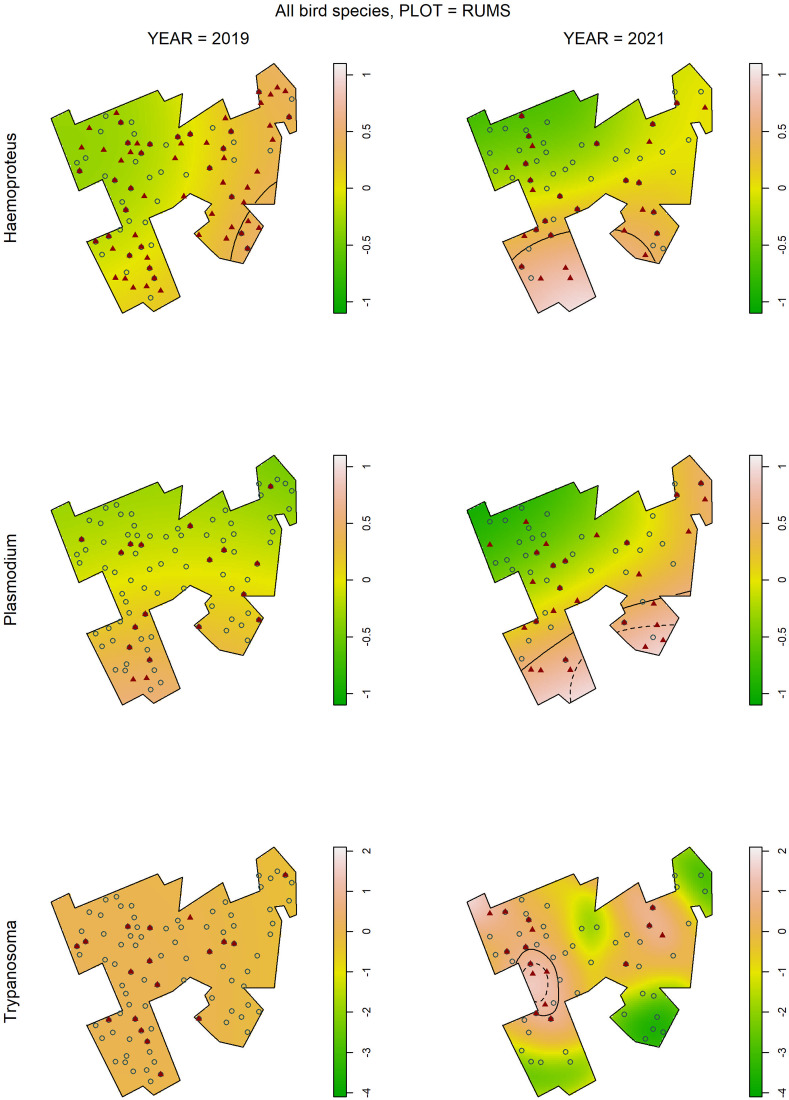
Kernel-smoothed maps showing log-relative risk of infection with *Haemoproteus*, *Plasmodium* and *Trypanosoma* in each year of the study, across the biggest study plot (Rums) for all bird species. Value ranges (depicted by colours) have been scaled to the maximum and minimum values observed across both years separately for each parasite genus. Contour lines depict areas of significantly elevated relative risk (solid line for *P* < 0.05; dashed line for *P* < 0.01). The locations of infected individuals are shown by red filled triangles, uninfected ones by open circles.

In general, in the majority of maps depicting the relative risk (odds) of infection with either *Haemoproteus*, *Plasmodium* or *Trypanosoma* (30 plot × parasite genus × year combinations), we observed some spatial variation in the risk (Fig. 4 and Supplementary Fig. S2). However, only in a few maps, did we identify areas within plots with a significantly elevated risk of infection (*P* < 0.05, and in some cases *P* < 0.01; marked in the maps as contour lines). Overall, within-plot spatial infection risks for either *Haemoproteus*, *Plasmodium* or *Trypanosoma* varied temporally, i.e. no common pattern was observed in both study years. Only in the case of *Haemoproteus* infection in 1 plot (Rums) and *Plasmodium* infections in 2 plots (Rums, Balshage) was spatial variation in infection risk generally repeatable in 2019 and 2021. In the largest plot (Rums), we observed a north–south or northwest–southeast gradient of infection risk for *Haemoproteus* and *Plasmodium* parasites, which coincided with the areas of significantly elevated infection risk (*P* < 0.05, and in some cases *P* < 0.01).

## Discussion

We show that in 3 forest-dwelling passerine species, the risk of infection with common blood parasites – either *Haemoproteus*, *Plasmodium* or *Trypanosoma* – is shaped by a set of different parameters. These interspecific differences arise despite the fact that all 3 host species utilize the same breeding area, the same nesting niche and were sampled at the same time point of their yearly cycle – late nesting period. Depending on the identity of the host species and the parasite taxon, infection risk was associated with either body condition (with contrasting effects for *Haemoproteus* and *Plasmodium* parasites), host density or one of the landscape metrics. In no case did we find that a single parameter explained variation in infection risk with parasites from each genus in more than 1 host species. Importantly, we found in one of the 2 resident host species that the risk of infection with *Plasmodium* was related to the distance to the land type used for agricultural activities. Spatial within-plot variation in infection risk with parasites from each genus showed limited repeatability across time, and infection risk gradients overlapping areas with significantly elevated infection risks were found only in the largest plot.

### Prevalence and diversity of blood parasites

The prevalence of blood parasites varied among the study host species, with the smallest differences observed in infections with *Trypanosoma* and the highest in infections with *Plasmodium*. In comparison with previous studies on the same host species in the study area, we found a much higher prevalence of *Haemoproteus* in all bird species, reaching 47.8% in blue tits compared to previously reported rates of 15.7% (Podmokła *et al*., 2014), 72.7% in great tits compared to 46.3% (Dubiec *et al*., 2016) and 36% in collared flycatchers compared to 29.7% (Radwan *et al*., 2012) or 17.4% (Fletcher *et al*., 2019). Conversely, infections caused by *Plasmodium* showed comparable prevalence in collared flycatchers (4% in our study and previously reported rates of 4.7 and 4.5%, respectively), but much lower prevalence in both tit species (blue tits: 26.9% in our study compared to 46.6% previously reported; great tits: 44.4% in our study compared to 78.4% previously reported). Such comparisons were not possible for *Trypanosoma* infections, because earlier studies did not include parasites from this genus. The temporal variation in infection rates with avian haemoparasites that we found in our host–parasite network is commonly reported (Bensch *et al*., 2007; Podmokła *et al*., 2014), and may be attributed to changes in vector abundance, especially given the approximately 10-year gap between these studies. Such fluctuations are also evident at the much shorter time window, as shown by the current study when large differences were detected over the span of 2 years.

In comparison with previous studies, we detected similar number of *Haemoproteus* lineages in all 3 hosts and fewer *Plasmodium* lineages in great tits and collared flycatchers (Radwan *et al*., 2012; Podmokła *et al*., 2014; Dubiec *et al*., 2016; Fletcher *et al*., 2019). These differences may be attributed to the smaller sample sizes of screened individuals of each bird species in the current (blue tits: 64, great tits: 275, collared flycatchers: 267) compared to previous studies (blue tits: 762, great tits: 324, collared flycatchers: 212 and 512), as the number of detected lineages asymptotically increases with the number of screened individuals (Neto *et al*., 2015). Additionally, the primers used for amplifying *Haemoproteus* and *Plasmodium* sequence fragments (Yeo *et al*., 2022) did not allow for the distinction of certain parasitic lineages in some cases (refer to Results), which may have contributed to a reduction in the number of detected lineages. On the other hand, the NGS used in the present study enhances the detectability of multiple parasites when the host carries 2 or more lineages (Jarvi *et al*., 2013; Galen *et al*., 2020). Moreover, the high sensitivity of NGS allows the identification of parasitic lineages with very low infection intensities, which may be undetectable using Sanger sequencing. For example, we unexpectedly found lineage hCOLL3 in blue tits and great tits. Although these bird species are not typical hosts for this parasite, the transmission of this parasitic lineage was already confirmed in juvenile collared flycatchers on Gotland (Fletcher *et al*., 2019). Additionally, the sequence counts of hCOLL3 were much lower in tits (blue tits: range: 1–57, mean: 13; great tits: range: 1–54, mean: 12, Supplementary Table S2) than in collared flycatchers, which are its typical hosts (range: 1–1436, mean: 249). Assuming that the sequence counts of the lineage might be related to the intensity of infection, these low counts in tits might suggest that *Haemoproteus balmonari* hCOLL3 does not complete its development in those hosts (Valkiūnas *et al*., 2009). We may not, however, exclude the possibility that the spectrum of hosts for this parasite has expanded over the past 10 years (the period since previous studies). The other unexpected lineages, hARW1 and hRW2, were detected in all 3 bird species. Both lineages are specific to warblers (Acrocephalidae), and hRW2 was already detected in juveniles in Sweden (Ellis *et al*., 2020) and Bulgaria (Dimitrov *et al*., 2018), confirming the possibility of its transmission in Europe. Their prevalence in tits and collared flycatchers does not exceed 3.5% (Table 2), and their sequence counts reach a maximum of 26 (Supplementary Table S2). This suggests that these infections might be cases of abortive haemosporidian infections that probably would not be detected using traditional sequencing methods.

### Individual traits

We identified relationships between body condition and the probability of infection with haemosporidians. In the great tit, better condition increased the likelihood of *Haemoproteus* infection, whereas in blue tits, better condition decreased the likelihood of *Plasmodium* infection. These contrasting directions may be associated with differences in the pathogenicity of the respective genera. *Plasmodium* is considered a more pathogenic parasite than *Haemoproteus* (Valkiūnas, 2005). Consequently, *Plasmodium* infection may reduce condition, as well as individuals with lower condition may be more susceptible to new infections or reactivation of latent infections. In contrast, in the case of *Haemoproteus*, infection intensities remain higher than in *Plasmodium* infections (Podmokła *et al*., 2014), and their consequences are milder (Atkinson *et al*., 2008). This may allow for the maintenance of good condition even in the presence of infection. However, the increase in the likelihood of *Haemoproteus* infection with improving condition must be associated with additional factors. It is, for example, possible that individuals in better condition occupy habitats superior in terms of availability and quality of food, but which simultaneously support a high abundance of arthropods vectoring *Haemoproteus*. The observed relationship may also result from parasite-mediated selection (Goater and Holmes, 1997), where infected individuals might have survived an acute stage of infection and, in fact, be of superior quality, enabling them to better cope with the consequences of harbouring the infection. A negative or positive relationship between the infection probability with haemosporidians and host body condition has been reported in many studies, but the lack of such association seems to be equally common (e.g. Jiménez-Peñuela *et al*., 2019; Santiago-Alarcon *et al*., 2019; Gutiérrez-Ramos *et al*., 2024).

The other individual traits, such as age and sex, did not show any influence on the probability of infection with any of the studied parasitic genera. This is unexpected given that sex and especially age were identified as predictors of haemoparasite infection risks in many avian populations (e.g. Wood *et al*., 2007; Valdebenito *et al*., 2023).

### Population and landscape metrics

The association between avian blood parasite prevalence and population and landscape metrics varied among host species. In the case of population metrics, *Haemoproteus* infection was positively related to conspecific density in great tits, whereas in collared flycatchers, it related to all bird species, indicating enhanced transmission within denser populations, consistent with previous studies (Ortego and Cordero, 2010; Lachish *et al*., 2013; Ferraguti *et al*., 2018). A positive link between host density and infection rate could also result from indirect effects, such as elevated stress compromising immune function or reduced self-maintenance time due to high rates of interaction with conspecifics.

In the case of landscape metrics, our models indicated a positive relationship between the distance to pastures and *Plasmodium* infections in blue tits. This finding was unexpected as we anticipated that pasture areas would provide favourable conditions for vectors due to the food reservoir provided by grazing animals. It is possible that the presence of livestock meets the dietary needs of vectors, resulting in lower infection probabilities in their vicinity and increasing with distance from pastures. Similarly, regarding another variable related to human activity, namely the distance to agricultural fields, our predictions did not hold true. We did not detect any association between the probability of infection and the distance to fields for any host species or parasite genus. It is possible that the expected negative impact of agricultural fields on vectors does not occur in the studied area, possibly due to limited pesticide use, as Gotland primarily practices organic farming (Ekroth, 2021). Another variable indirectly related to human activity is NDMI, as land use can affect snow retention or faster drying of soil. Our analyses showed a positive relationship between NDMI and *Haemoproteus* infections in blue tits, potentially indicating favourable conditions for vector breeding and parasite transmission in moister areas. Another landscape metric potentially influencing vectors is proximity to the coast. We found a positive relationship between proximity to the coast and *Trypanosoma* infections in collared flycatchers, likely due to reduced host-seeking activity of vectors in high-wind coastal areas (Martínez-De La Puente *et al*., 2009).

Relative risk maps revealed that only a few combinations of plot, parasite taxon and year showed significantly elevated infection risk areas, and their occurrence and overall spatial variation were hardly consistent over 2 years. This result indicates that some temporally variable factors, such as climatic conditions, which in turn may affect vector population size, may best explain spatial variation in infection risk. This contrasts with Lachish *et al*. (2013), who found consistent spatial infection risk in blue and great tits in the Wytham Woods (UK).

Overall, our study provides valuable insights into the ecological drivers of avian blood parasite dynamics, emphasizing the need for comprehensive landscape-level approaches to understand and mitigate disease transmission in natural ecosystems.

## Supporting information

Podmokła et al. supplementary material Podmokła et al. supplementary material

## Data Availability

The dataset used in this study is available on Zenodo: https://zenodo.org/doi/10.5281/zenodo.12701531.

## Supplementary material

The supplementary material for this article can be found at https://doi.org/10.1017/S0031182024000994.
