# Supplementary material for: Haemoparasite infection risk in multi-host avian system: an integrated analysis
Source: Parasitology. 2024 Nov 20;151(11):1242–53. doi: 10.1017/S0031182024000994 (PMC11894008; doi:10.1017/S0031182024000994)
Supplement: Podmokła et al. supplementary material [file S0031182024000994sup001.pdf]

Supplementary Information

Haemoparasite Infection Risk in Multi-Host Avian System: An Integrated Analysis

Edyta Podmokła, Anna Dubiec, Bartosz Pluciński, Bartłomiej Zając, Lars Gustafsson

Correspondence: edyta.podmokla@uj.edu.pl

Appendix S1. Details on designing primers for *Trypanosoma*

Table S1. Primers sequences used for NGS sequencing

Table S2. Detailed information on observed sequence counts for each parasite lineage and bird species

Appendix S2. Methodological details on: A) calculation landscape and population metrics; B) NDVI and NDMI estimation; C) creating maps with relative risk of infection

Table S3. Detailed information on specific distances for each bird species

Figure S1. Correlation matrix of the landscape and population variables

Table S4. Results of Collinearity Diagnostics for general models examining the effects of the individual variables on the status of infection

Table S5. Results of Collinearity Diagnostics for general models examining the effects of the landscape and population variables on the status of infection

Table S6. AICc-ranked candidate models (within ΔAICc < 4) examining the effects of individual traits on the status of infection

Table S7. AICc-ranked candidate models (within ΔAICc < 4) examining the effects of the landscape and population variables on the status of infection

Figure S2. Kernel-smoothed maps showing log-relative risk of infections

Appendix S1. Designing primers for *Trypanosoma.*

Primers were designed based on sequences from Zídková et al. (2012). We downloaded all *Trypanosoma* sequences amplified in birds (a total of 36, see Table 1. from Zídková et al. 2012), aligned them, and identified variable regions (for potential amplicons) and conserved region (for primer sequences). After designing several primer pairs using OligoPerfect from Thermofisher and testing them in the lab, we selected the pair T918F and T1296R, where the numbers indicate the position in the alignment of all sequences relative to *Trypanosoma* sp. LZ-2011 isolate PAS96 18S ribosomal RNA gene partial sequence, accession number: JN006848 (refer to Fig. S1).

Table 1. from Zídková et al. 2012, with sequences used for designing primers, highlighted in yellow.

Isolate	Host species (scientific name)	Host species (English typical name)	Year	Origin	GenBank Acc. no.	Isolation	Used for primer designing
A1412	*Corvus frugilegus*	Rook	1978	Krkonoše mountains (CZ)	U39578	Bedrník	1
AGE3	*Accipiter gentilis*	Goshawk/adult	2002	Milovice game preserve (CZ)	JN006829	Votýpka et al.	1
ANI14A	*Accipiter nisus*	Sparrowhawk/nestling	1999	Prague (CZ)	AY099318	Votýpka et al.	1
ANI14B	*Accipiter nisus*	Sparrowhawk/nestling	1999	Prague (CZ)	FJ649483	Votýpka et al.	1
ANI21	*Accipiter nisus*	Sparrowhawk/nestling	2001	Prague (CZ)	RAPD only	Votýpka et al.	
ANI36	*Accipiter nisus*	Sparrowhawk/nestling	2001	Prague (CZ)	RAPD only	Votýpka et al.	
ANI54	*Accipiter nisus*	Sparrowhawk/nestling	2001	Prague (CZ)	JN006849	Votýpka et al.	1
APO1	*Aquila pomarina*	Lesser spotted eagle/nestling	1997	Vyšné Ružbachy (SK)	AF416559	Votýpka et al.	1
APO7	*Aquila pomarina*	Lesser spotted eagle/nestling	2000	Vyšné Ružbachy (SK)	JF778738	Votýpka et al.	1
T. carrassi BD	*Cyprinus carpio*	Common carp	1974	Českobudějovicko (CZ)	RAPD only	Lom	
BUT15	*Buteo buteo*	Buzzard/nestling	1999	Milovice game preserve (CZ)	AY099320	Votýpka et al.	1
BUT16	*Buteo buteo*	Buzzard/nestling	1999	Milovice game preserve (CZ)	RAPD only	Votýpka et al.	
BUT17	*Buteo buteo*	Buzzard/nestling	1999	Prague (CZ)	JN006854	Votýpka et al.	1
BUT19	*Buteo buteo*	Buzzard/nestling	1999	Prague (CZ)	JN006828	Votýpka et al.	1
BUT23	*Buteo buteo*	Buzzard/nestling	2000	Prague (CZ)	RAPD only	Votýpka et al.	
BUT26	*Buteo buteo*	Buzzard/nestling	2000	Prague (CZ)	RAPD only	Votýpka et al.	
BUT50	*Buteo buteo*	Buzzard/nestling	2001	Prague (CZ)	JN006825	Votýpka et al.	1
CUL1	*Culex pipiens*	Northern house mosquito	1998	Milovice game preserve (CZ)	AF416561	Votýpka et al.	
CUL2	*Culex pipiens*	Northern house mosquito	1999	Milovice game preserve (CZ)	JN006834	Votýpka et al.	
CUL5	*Culex pipiens*	Northern house mosquito	2000	Milovice game preserve (CZ)	JN006838	Votýpka et al.	
CUL6	*Culex pipiens*	Northern house mosquito	2000	Milovice game preserve (CZ)	HQ107970	Votýpka et al.	
CUL15	*Culex pipiens*	Northern house mosquito	2000	Prague (CZ)	JN006830	Votýpka et al.	
CUL24	*Culex pipiens*	Northern house mosquito	2001	Prague (CZ)	RAPD only	Votýpka et al.	
CUL28	*Culex pipiens*	Northern house mosquito	2002	Třeboňsko (CZ)	HQ107967	Votýpka et al.	
CUL30	*Culex modestus*	mosquito	2002	Třeboňsko (CZ)	HQ909084	Votýpka et al.	
CUL31	*Culex pipiens*	Northern house mosquito	2006	Třeboňsko - Ruda (CZ)	HQ107968	Votýpka	
FT2	*Falco tinnunculus*	Kestrel/adult	1999	Slatina nad Zdobnicí (CZ)	AY099319	Votýpka et al.	1

T. corvi ITMAP180795	*Corvus frugilegus*	Rook	1970	Fordingbridge, Hants (GB)	AY461665	Baker	1
OA06	*Ornithomyia avicularia*	Hippoboscid fly	1999	Prague (CZ)	AF416562	Votýpka et al.	
OA08	*Ornithomyia avicularia*	Hippoboscid fly	2000	Prague (CZ)	JN006844	Votýpka et al.	
OA11	*Ornithomyia avicularia*	Hippoboscid fly	2001	Prague (CZ)	JN006824	Votýpka et al.	
OA12	*Ornithomyia avicularia*	Hippoboscid fly	2001	Prague (CZ)	RAPD only	Votýpka et al.	
PAS21	*Fringilla coelebs*	Chaffinch/1st year juvenile	2002	Milovice game preserve (CZ)	JN006826	Svobodová et al.	1
PAS23	*Emberiza citrinella*	Yellowhammer/adult	2002	Milovice game preserve (CZ)	JN006850	Svobodová et al.	1
PAS44	*Sitta europaea*	Nuthatch/1st year juvenile	2003	Milovice game preserve (CZ)	JN006837	Svobodová et al.	1
PAS48	*Sylvia atricapilla*	Blackcap/1st year juvenile	2003	Milovice game preserve (CZ)	JN006845	Svobodová et al.	1
PAS56	*Fringilla coelebs*	Chaffinch/adult	2004	Milovice game preserve (CZ)	JN006827	Svobodová et al.	1
PAS64	*Emberiza citrinella*	Yellowhammer/adult	2004	Milovice game preserve (CZ)	JN006851	Svobodová et al.	1
PAS71	*Turdus philomelos*	Song thrush/adult	2004	Milovice game preserve (CZ)	JN006847	Svobodová et al.	1
PAS72	*Parus caeruleus*	Blue tit/yearling	2004	Milovice game preserve (CZ)	JN006846	Svobodová et al.	1
PAS93	*Ficedula albicollis*	Collared flycatcher/adult	2006	Pohansko (CZ)	JN006852	Svobodová et al.	1
PAS94	*Ficedula albicollis*	Collared flycatcher/adult	2006	Pohansko (CZ)	JN006841	Svobodová et al.	1
PAS95	*Ficedula albicollis*	Collared flycatcher/adult	2006	Milovice game preserve (CZ)	JN006842	Svobodová et al.	1
PAS96	*Ficedula albicollis*	Collared flycatcher/adult	2006	Milovice game preserve (CZ)	JN006848	Svobodová et al.	1
PAS99	*Ficedula albicollis*	Collared flycatcher/adult	2006	Milovice game preserve (CZ)	HQ107969	Svobodová et al.	1
PAS105	*Phylloscopus collybita*	Chiffchaff/adult	2007	Milovice game preserve (CZ)	JN006831	Svobodová et al.	1
PAS106	*Phylloscopus collybita*	Chiffchaff/adult	2007	Milovice game preserve (CZ)	JN006833	Svobodová et al.	1
PAS107	*Ficedula albicollis*	Collared flycatcher/adult	2007	Pohansko (CZ)	JN006835	Svobodová et al.	1
PAS108	*Ficedula albicollis*	Collared flycatcher/adult	2007	Pohansko (CZ)	JN006840	Svobodová et al.	1
PAS109	*Ficedula albicollis*	Collared flycatcher/adult	2007	Pohansko (CZ)	HQ107966	Svobodová et al.	1
PAS110	*Phylloscopus collybita*	Chiffchaff/adult	2007	Milovice game preserve (CZ)	JN006836	Svobodová et al.	1
PAS111	*Ficedula albicollis*	Collared flycatcher/adult	2007	Pohansko (CZ)	JN006843	Svobodová et al.	1
PAS112	*Phylloscopus collybita*	Collared flycatcher/adult	2007	Milovice game preserve (CZ)	JN006832	Svobodová et al.	1
PAS113	*Phylloscopus collybita*	Chiffchaff/adult	2007	Milovice game preserve (CZ)	JN006839	Svobodová et al.	1
PAS114	*Phylloscopus sibilatrix*	Wood warbler/adult	2007	Milovice game preserve (CZ)	JN006853	Svobodová et al.	1
SIM1	*Eusimulium latipes*	Black fly	1999	Milovice game preserve (CZ)	RAPD only	Votýpka et al.	
SIM3	*Eusimulium securiforme*	Black fly	1999	Milovice game preserve (CZ)	AF416563	Votýpka et al.	
SIM4	*Eusimulium securiforme*	Black fly	1999	Prague (CZ)	RAPD only	Votýpka et al.	
SIM6	*Eusimulium securiforme*	Black fly	1999	Prague (CZ)	RAPD only	Votýpka et al.	
SIM8	*Eusimulium securiforme*	Black fly	2000	Milovice game preserve (CZ)	RAPD only	Votýpka et al.	
SIM13	*Eusimulium securiforme*	Black fly	2001	Milovice game preserve (CZ)	RAPD only	Votýpka et al.	
SIM17	*Eusimulium securiforme*	Black fly	2001	Milovice game preserve (CZ)	RAPD only	Votýpka et al.	
SIM28	*Eusimulium securiforme*	Black fly	2002	Prague (CZ)	RAPD only	Votýpka et al.	
T.brucei STIB247	*Alcelaphus buselaphus cokii*	Coke’s hartebeest	1971	Serengeti Area (TZ)	RAPD only	Geigy and Kauffman	
Wallaceina inconstans	Grypocoris sexguttatus	Bug	1986	Pskov region (RS)	RAPD only	Podlipaev et al.	

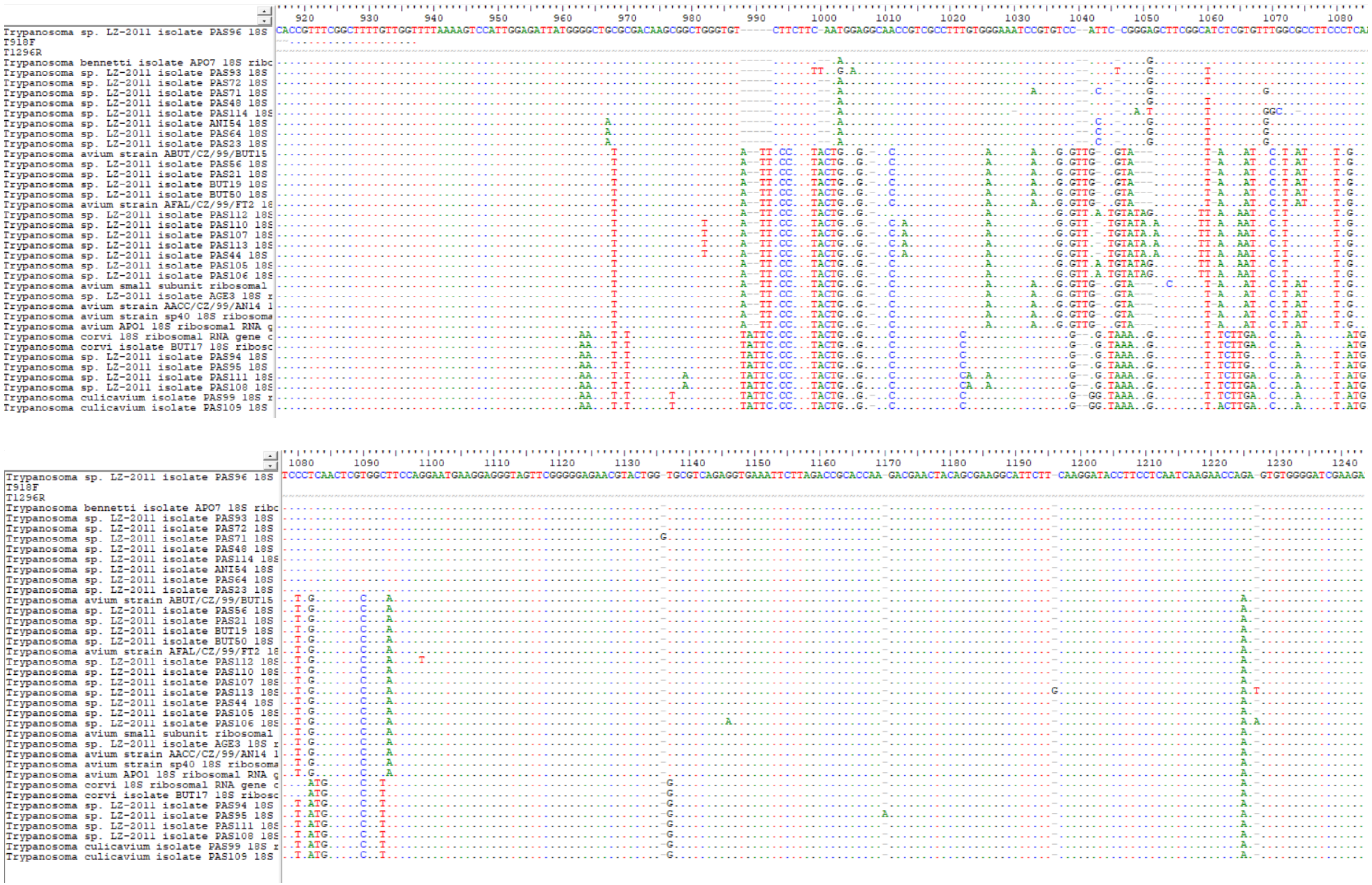
920 930 940 950 960 970 980 990 1000 1010 1020 1030 1040 1050 1060 1070 1080
Trypanosoma sp. LZ-2011 isolate PAS96 18S
T918F
T1296R
Trypanosoma bennetti isolate APO7 18S ribo
Trypanosoma sp. LZ-2011 isolate PAS93 18S
Trypanosoma sp. LZ-2011 isolate PAS72 18S
Trypanosoma sp. LZ-2011 isolate PAS71 18S
Trypanosoma sp. LZ-2011 isolate PAS48 18S
Trypanosoma sp. LZ-2011 isolate PAS114 18S
Trypanosoma sp. LZ-2011 isolate ANI54 18S
Trypanosoma sp. LZ-2011 isolate PAS64 18S
Trypanosoma sp. LZ-2011 isolate PAS23 18S
Trypanosoma avium strain ABUT/CZ/99/BUT15
Trypanosoma sp. LZ-2011 isolate PAS56 18S
Trypanosoma sp. LZ-2011 isolate PAS21 18S
Trypanosoma sp. LZ-2011 isolate BUT19 18S
Trypanosoma sp. LZ-2011 isolate BUT50 18S
Trypanosoma avium strain AFAL/CZ/99/FT2 18
Trypanosoma sp. LZ-2011 isolate PAS112 18S
Trypanosoma sp. LZ-2011 isolate PAS110 18S
Trypanosoma sp. LZ-2011 isolate PAS107 18S
Trypanosoma sp. LZ-2011 isolate PAS113 18S
Trypanosoma sp. LZ-2011 isolate PAS44 18S
Trypanosoma sp. LZ-2011 isolate PAS105 18S
Trypanosoma sp. LZ-2011 isolate PAS106 18S
Trypanosoma avium small subunit ribosomal
Trypanosoma sp. LZ-2011 isolate AGE3 18S r
Trypanosoma avium strain AACC/CZ/99/AN14 1
Trypanosoma avium strain sp40 18S ribosoma
Trypanosoma avium APO1 18S ribosomal RNA g
Trypanosoma corvi 18S ribosomal RNA gene c
Trypanosoma corvi isolate BUT17 18S ribosc
Trypanosoma sp. LZ-2011 isolate PAS94 18S
Trypanosoma sp. LZ-2011 isolate PAS95 18S
Trypanosoma sp. LZ-2011 isolate PAS111 18S
Trypanosoma sp. LZ-2011 isolate PAS108 18S
Trypanosoma culicavium isolate PAS99 18S r
Trypanosoma culicavium isolate PAS109 18S
1080 1090 1100 1110 1120 1130 1140 1150 1160 1170 1180 1190 1200 1210 1220 1230 1240

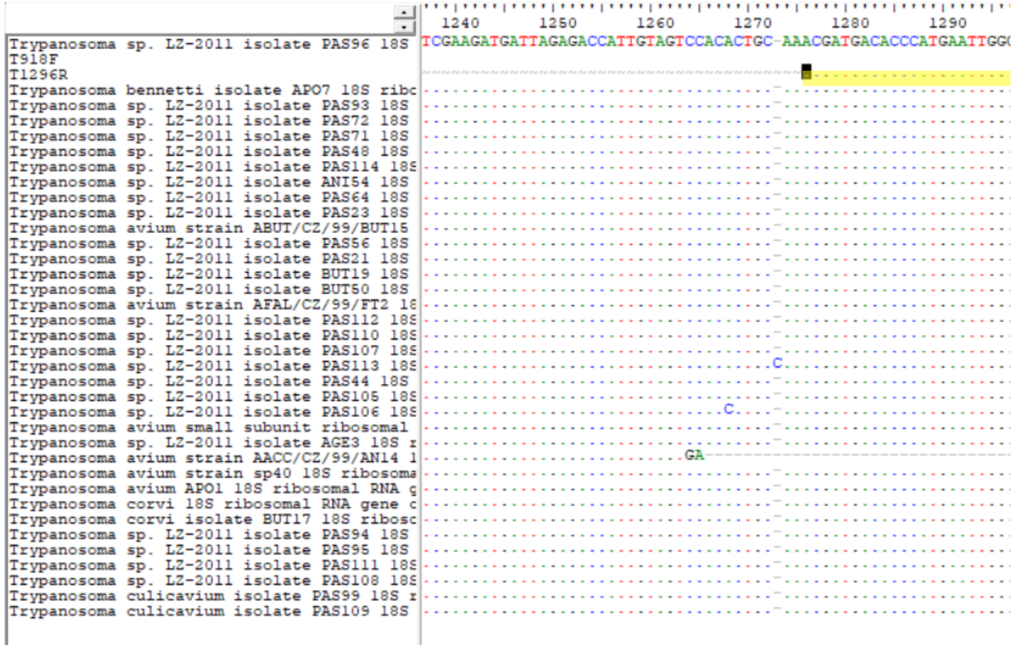

Fig. 1. Location of the designed primers on the alignment of all *Trypanosoma* sequences from Zídková et al. (2012)

References

Zídková, L., Cepicka, I., Szabová, J. and Svobodová, M. (2012). Biodiversity of avian trypanosomes. *Infection, Genetics and Evolution* **12**, 102–112. doi: 10.1016/j.meegid.2011.10.022.

Table S1. Primers sequences used for NGS sequencing. *Haemoproteus* and *Plasmodium* primers amplified a part of mitochondrial cytochrome b gene, whereas *Trypanosoma* primers amplified a part of the small subunit ribosomal RNA (SSU rRNA) gene.

Target genus	Primers	Sequences (5´-3´)	Amplicon size (bp) with (without primers)
Haemoproteus & Plasmodium	HaemNF1[a]	CATATATTAAGAGAAITATGGAG	408 (361)
	HaemNRShort[b]	GATTAGAGCTACCTTGTAAATGTA	
Trypanosoma	T918F	CCGTTTCGGCTTTTGTTGGT	365-379 (324-338)
	T1296R	CCAATTCATGGGTGTCATCGT	

[a] Hellgren et al. (2004)

[b] Yeo et al. (2022)

The unique primer-tag, consisting of a combination of 6 nucleotides, was attached to the above primers, allowing for the indexing of individual samples. An example of the primer sequence used is: NAACCGACATATATTAAGAGAAITATGGAG, where N enables the ligation of Illumina adapters in a later step; AACCGA is the unique tag; CATATATTAAGAGAAITATGGAG is the region of interest-specific primer (HaemNF1 from above table). We used 16 different tags joined to the forward primers and 12 tags to the reverse primers. The primers were applied with a multichannel pipette on a PCR plate, allowing for the unique indexing of 192 samples, forming a single library (2 PCR plates × 96 wells; on each plate 8 rows = 8 forward tags and 12 columns = 12 reverse tags).

References

Hellgren, O., Waldenström, J. and Bensch, S. (2004). A new PCR assay for simultaneous studies of Leucocytozoon, Plasmodium, and Haemoproteus from avian blood. *Journal of Parasitology* **90**, 797–802. doi: 10.1645/GE-184R1.

Yeo, H., Harjoko, D. N. and Rheindt, F. E. (2022). Double trouble: untangling mixed sequence signals in bird samples with avian haemosporidian co-infections. *Parasitology* **149**, 799–810. doi: 10.1017/S0031182022000245.

Table S2. Detailed information on observed sequence counts for each parasite lineage and bird species.

	All species					Collared flycatcher					Blue tit & Great tit					Blue tit					Great tit				
	nbr.val	min	max	median	mean	nbr.val	min	max	median	mean	nbr.val	min	max	median	mean	nbr.val	min	max	median	mean	nbr.val	min	max	median	mean
**H_ARW1_Haemoproteus_belopolskyi**	22	2	22	10.50	10.91	11	2	22	9	8.73	11	6	22	13	13.09	2	7	17	12	12.00	9	6	22	13	13.33
**H_COLL2_Haemoproteus_pallidus**	22	1	366	1.00	42.55	15	1	366	3	61.93	7	1	1	1	1.00	2	1	1	1	1.00	5	1	1	1	1.00
**H_COLL3_Haemoproteus_balmorali**	243	1	1436	7.00	105.57	96	1	1436	5.5	248.93	147	1	57	7	11.95	32	1	57	8.5	13.44	115	1	54	7	11.54
**H_PARUS1**	265	1	1163	8.00	187.77	36	1	5	1	1.50	229	1	1163	48	217.05	39	1	1163	80	242.82	190	1	1092	47.5	211.76
**H_PARUS68**	8	1	7	2.50	3.38	4	2	7	4	4.25	4	1	5	2	2.50	1	5	5	5	5.00	3	1	3	1	1.67
**H_PHSIB1_Haemoproteus_majoris**	333	1	1146	37.00	122.60	99	1	553	7	66.31	234	1	1146	50	146.42	26	1	47	2	3.88	208	1	1146	73.5	164.24
**H_RW2**	26	1	26	10.00	10.85	13	1	25	10	10.92	13	2	26	10	10.77	3	8	11	9	9.33	10	2	26	10	11.20
**H_SFC1_Haemoproteus_balmorali**	12	1	2	1.00	1.08						12	1	2	1	1.08	2	1	2	1.5	1.50	10	1	1	1	1.00
**H_WW2_Haemoproteus_majoris**	115	1	426	3.00	11.24	3	1	2	1	1.33	112	1	426	3	11.51	9	1	8	2	2.67	103	1	426	3	12.28
**P_BT7**	105	1	473	4.00	18.54	4	1	5	3	3.00	101	1	473	4	19.16	13	1	115	1	27.77	88	1	473	4	17.89
**P_COLL7**	3	1	44	3.00	16.00	3	1	44	3	16.00															
**P_GRW09**	9	1	11	2.00	2.89	1	11	11	11	11.00	8	1	5	1.5	1.88	2	1	1	1	1.00	6	1	5	2	2.17
**P_GRW11_Plasmodium_relictum**	24	1	91	4.50	12.08	1	1	1	1	1.00	23	1	91	5	12.57						23	1	91	5	12.57
**P_LAMPUR03**	1	61	61	61.00	61.00	1	61	61	61	61.00															
**P_PLOPRI01**	2	92	209	150.50	150.50	2	92	209	150.5	150.50															
**P_RTSR1**	10	1	262	1.00	27.40	2	2	262	132	132.00	8	1	3	1	1.25	1	1	1	1	1.00	7	1	3	1	1.29
**P_SGS1_Plasmodium_relictum**	56	1	602	4.50	44.86	5	1	60	1	13.20	51	1	602	5	47.96	6	1	19	3.5	7.33	45	1	602	5	53.38
**P_SW2_Plasmodium_homonucleophilum**	147	1	1972	3.00	180.46	25	1	4	1	1.28	122	1	1972	16.5	217.17	7	1	60	1	9.57	115	1	1972	45	229.81
**P_TURDUS1_Plasmodium_circumflexum**	146	1	239	5.00	14.93	3	1	3	2	2.00	143	1	239	5	15.20	27	1	219	6	20.52	116	1	239	5	13.97
**P_WW4**	3	27	239	196.00	154.00	3	27	239	196	154.00															
**Trypanosoma_avium_APO1**	32	1	175	18.00	45.66	13	8	162	78	78.23	19	1	175	10	23.37	4	6	79	14.5	28.50	15	1	175	8	22.00
**Trypanosoma_avium_small_subunit_ribosomal_RNA**	117	1	48	3.00	4.32	6	1	48	5	11.67	111	1	17	3	3.92	19	1	8	3	3.26	92	1	17	3	4.05
**Trypanosoma_bennetti_isolate_APO7**	127	1	125	2.00	5.84	12	1	125	10.5	34.83	115	1	12	2	2.82	22	1	6	3	2.95	93	1	12	2	2.78
**Trypanosoma_corvi_isolate_BUT17**	1	40	40	40.00	40.00	1	40	40	40	40.00															
**Trypanosoma_culicavium_isolate_PAS109**	10	2	83	21.00	31.20	10	2	83	21	31.20															
**Trypanosoma_culicavium_isolate_PAS99**	72	1	223	3.00	18.22	33	1	223	26	37.52	39	1	12	1	1.90	8	1	12	1	2.88	31	1	6	1	1.65
**Trypanosoma_sp._LZ.2011_isolate_PAS105**	20	1	611	2.00	71.10	6	12	611	160.5	233.00	14	1	4	1	1.71	3	1	4	2	2.33	11	1	4	1	1.55
**Trypanosoma_sp._LZ.2011_isolate_PAS108**	10	2	159	21.50	48.10	10	2	159	21.5	48.10															
**Trypanosoma_sp._LZ.2011_isolate_PAS23**	64	1	111	2.00	7.73	9	1	111	15	31.22	55	1	37	2	3.89	10	1	25	2.5	6.80	45	1	37	2	3.24
**Trypanosoma_sp._LZ.2011_isolate_PAS44**	2	2	14	8.00	8.00	2	2	14	8	8.00															
**Trypanosoma_sp._LZ.2011_isolate_PAS48**	55	1	73	1.00	5.36	2	1	9	5	5.00	53	1	73	1	5.38	9	1	43	2	6.33	44	1	73	1	5.18
**Trypanosoma_sp._LZ.2011_isolate_PAS71**	101	1	223	2.00	12.03	6	4	74	29	31.83	95	1	223	2	10.78	21	1	223	2	25.10	74	1	67	2	6.72
**Trypanosoma_sp._LZ.2011_isolate_PAS93**	4	13	78	24.00	34.75	2	25	78	51.5	51.50	2	13	23	18	18.00	2	13	23	18	18.00					
**Trypanosoma_sp._LZ.2011_isolate_PAS95**	19	2	151	40.00	47.16	18	2	151	41.5	48.61	1	21	21	21	21.00						1	21	21	21	21.00
**Trypanosoma_sp._LZ.2011_isolate_PAS96**	16	1	43	1.00	3.94	1	43	43	43	43.00	15	1	2	1	1.33	2	2	2	2	2.00	13	1	2	1	1.23

Appendix S2. Methodological details on: A) calculation landscape and population metrics; B) NDVI and NDMI estimation; C) creating maps with relative risk of infection

A) Landscape and population metrics

The computation of distance and density parameters was conducted using Quantum GIS 3.28.12. Before initiating the calculations, we reprojected all relevant layers to the EPSG:3015 SWEREF99 18 45 coordinate reference system to ensure consistency in spatial analysis. The 'distance to nearest hub (points)' function was employed to determine the distance between each nestbox and the borders of: (i) the forest complex, providing a measure known as 'ForestEdge'; (ii) the nearest seacoast ('CoastEdge'); (iii) arable fields (‘Field’); (iv) pastures (‘Pasture’) (refer to Table S3, for detailed information on specific distances for each bird species). We used high resolution (10x10 m) Land Cover Map of Europe 2017 (Malinowski *et al.*, 2020) to designate and draw plots with different land uses, with additional corrections based on satellite images.

To assess potential host density, the 'Heatmap – Kernel Density Estimation' function was applied, utilizing a 56.4 m radius to calculate density per hectare. We set the pixel size to 10x10 m and employed a Quartic Kernel shape to capture the spatial distribution of potential hosts accurately. The input points for this density analysis were derived from the sampling data collected in each study year separately, allowing for a nuanced understanding of the density variations over time. It is essential to note that density indices were determined from nesting records rather than capture records, acknowledging that not all parents of active nests were captured in each year. Finally, we obtained indices of: (i) the local density of all bird species, providing a measure known as ‘AllSpecDens’, and (ii) the local density of individuals of the same species as the analysed species, providing a measure known as ‘OwnSpecDens’.

B) NDVI and NDMI estimation

For the calculation of Normalized Difference Vegetation Index (NDVI) and Normalized Difference Moisture Index (NDMI), we utilized all available Landsat 8 data products of Level 1, covering the study area during our research periods in April-June of 2019 and 2021. These products are radiometrically, atmospherically, and geometrically corrected satellite images at a 30 m spatial resolution. We carefully selected scenes with less than 80% cloud cover to ensure the accuracy of our analysis.

The selected Landsat 8 satellite images were downloaded, specifically extracting spectral bands with Red (band 4), Near-Infrared (NIR, band 5) and Short-Wave Infrared (SWIR, band 6). Subsequently, each raster layer was clipped to the area encompassing our research plots. The NDVI was then calculated using the standard formula: NDVI = (NIR - Red) / (NIR + Red), while NDMI = (NIR - SWIR) / (NIR + SWIR). EarthExplorer (https://earthexplorer.usgs.gov/) was employed for data retrieval, while processing and calculations were executed in the R environment using the 'raster' (Hijmans, 2023) and 'sf' (Pebesma and Bivand, 2023) packages.

Considering the uneven temporal sampling of available satellite images and concerns regarding data quality, primarily caused by cloud cover, we opted to limit our analysis to one NDVI and one NDMI raster layer per year of study. Specifically, for calculating NDVI we chose satellite images from the middle of June (17/06/2019 and 15/06/2021), capturing a representative snapshot of vegetation condition during the active period of adult vectors. For assessing NDMI, we decided to use images from the second half of April (23/04/2019 and 19/04/2021), due to very limited vegetation and quite high soil moisture caused by snowmelt, creating favourable conditions for the development of vector larvae.

C) Spatial variation in the relative risk of infection

To examine within-plot spatial variation in infection risk with parasites representing each of the three genera (pooled data for the three host species), we used maps of the relative risk, which depict the probability of infection relative to distribution of population at-risk. To generate maps, we used fixed-bandwidth kernel smoothing. To that end, we calculated the ratio of spatial densities generated by Gaussian kernel smoothing of the locations of infected and uninfected individuals. We estimated both densities with a jointly optimal fixed bandwidth using a joint selector proposed by Hazelton (2008). This approach, in order to find an optimal bandwidth, lends more weight to areas with more data. We also applied a shrinkage estimator of a lasso type to shrink a standard kernel estimator of the log-relative risk function towards zero (Hazelton, 2023). The use of shrinkage estimator may address the problem of unstable estimates of the relative risk in parts of the spatial window (plots in this case) with sparse data, while simultaneously maintaining necessary details in other parts. The tuning parameter λ (that controls the degree of shrinkage) for the lasso estimator was selected by cross-validation. Density estimates were corrected for edge effect bias using approach described by Jones (1993) and Diggle (2010). To identify areas of the plots with elevated risk of infection (at 0.05 and 0.01 level), *p*-value surfaces for shrinkage estimates of the log-relative risk function were calculated using the asymptotic approach (Hazelton and Davies, 2009). The analyses were carried out with an R package sparr (Davies *et al.*, 2018).

References

Davies, T. M., Marshall, J. C. and Hazelton, M. L. (2018). Tutorial on kernel estimation of continuous spatial and spatiotemporal relative risk. *Statistics in Medicine* **37**, 1191–1221. doi: 10.1002/sim.7577.

Diggle, P. J. (2010). Nonparametric methods. In *Handbook of Spatial Statistics* (ed. Gelfand, A. E., Diggle, P., Guttorp, P., and Fuentes, M.), pp. 299–316. CRC Press, Boca Raton, FL.

Hazelton, M. L. (2008). Kernel estimation of risk surfaces without the need for edge correction. *Statistics in medicine* **27**, 2269–2272. doi: 10.1002/SIM.3047.

Hazelton, M. L. (2023). Shrinkage estimators of the spatial relative risk function. *Statistics in Medicine* **42**, 4556–4569. doi: 10.1002/sim.9875.

Hazelton, M. L. and Davies, T. M. (2009). Inference based on kernel estimates of the relative risk function in geographical epidemiology. *Biometrical Journal* **51**, 98–109. doi: 10.1002/bimj.200810495.

Hijmans, R. J. (2023). raster: Geographic Data Analysis and Modeling. R package version 3.6-26, https://CRAN.R-project.org/package=raster.

Jones, M. C. (1993). Simple boundary correction for kernel density estimation. *Statistics and Computing* **3**, 135–146. doi: 10.1007/BF00147776/METRICS.

Malinowski, R., Lewiński, S., Rybicki, M., Gromny, E., Jenerowicz, M., Krupiński, M., Nowakowski, A., Wojtkowski, C., Krupiński, M., Krätzschmar, E. and Schauer, P. (2020). Automated Production of a Land Cover/Use Map of Europe Based on Sentinel-2 Imagery. *Remote Sensing 2020, Vol. 12, Page 3523* **12**, 3523. doi: 10.3390/RS12213523.

Pebesma, E. and Bivand, R. (2023). *Spatial Data Science: With Applications in R*. Chapman and Hall/CRC, New York doi: 10.1201/9780429459016.

Table S3. Detailed information on specific distances for each bird species.

	All species					Collared flycatche					Blue tit					Great tit				
**distance to:**	**n**	**min**	**max**	**median**	**mean**	**n**	**min**	**max**	**median**	**mean**	**n**	**min**	**max**	**median**	**mean**	**n**	**min**	**max**	**median**	**mean**
**ForestEdge**	609	3.8	155.0	52.0	57.7	267	5.7	155.0	52.6	56.3	67	13.7	130.1	49.4	56.6	275	3.8	146.2	52.6	59.3
**CoastEdge**	609	428.2	3002.3	1575.4	1648.7	267	428.2	3001.7	1657.1	1746.8	67	437.2	2930.8	1585.2	1665.3	275	440.1	3002.3	1538.4	1549.5
**Pasture**	609	0.3	403.0	94.2	130.9	267	0.3	403.0	81.4	123.3	67	23.0	403.0	110.9	160.0	275	0.4	403.0	97.6	131.1
**Field**	609	29.0	1439.6	352.4	565.9	267	31.8	1439.6	360.0	588.0	67	31.6	1344.6	364.5	556.7	275	29.0	1417.1	346.0	546.5

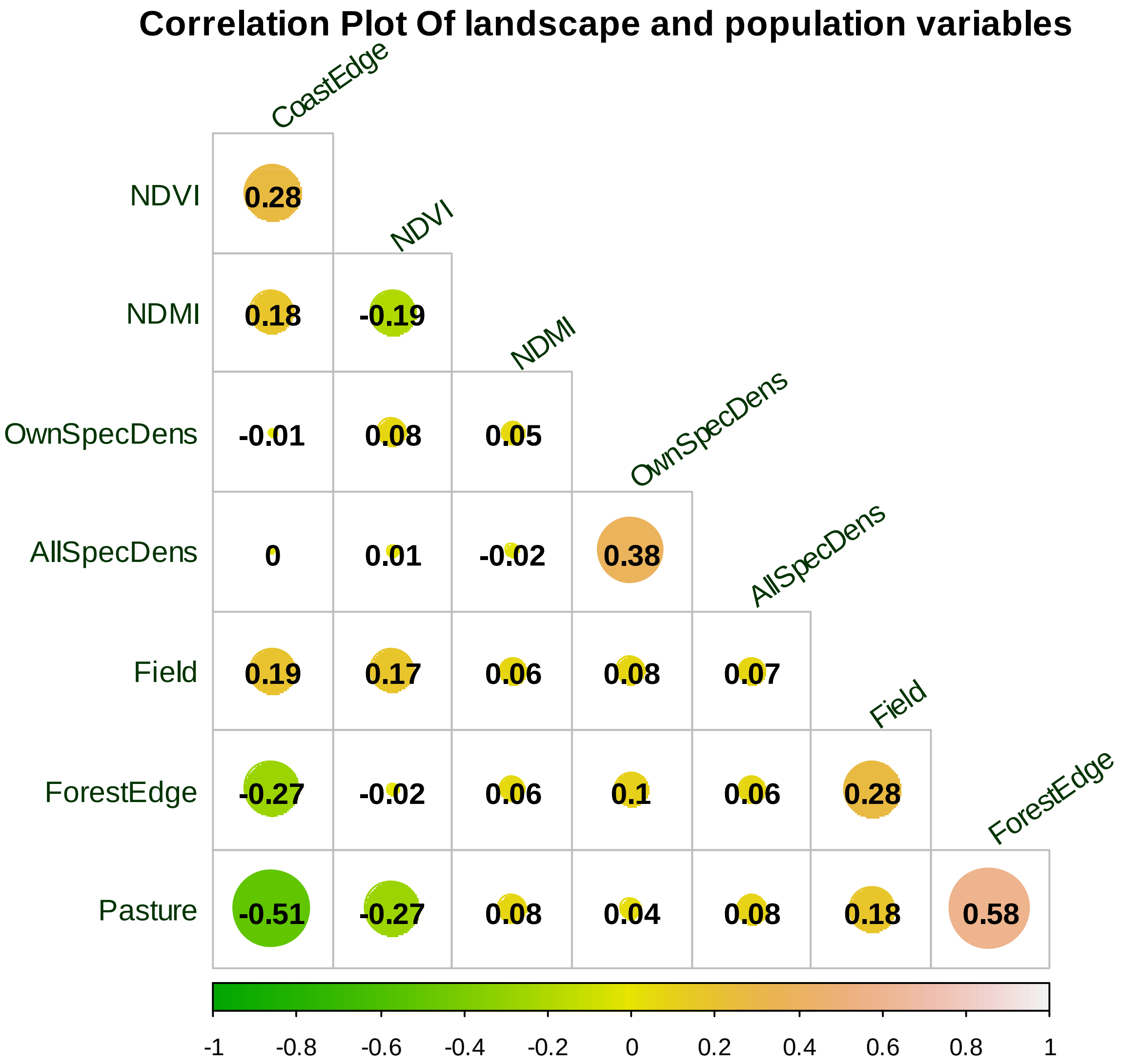

Figure S1. Correlation matrix of the landscape and population variables: woodland edge, coast edge, NDVI, NDMI, the local density of all bird species, the local density of the same species. The areas of circles show the value of corresponding Spearman correlation coefficients. Colour intensity (light to dark) and the size of the circle (small to big) are proportional to the correlation coefficients (0 to 1 for the positive coefficient and 0 to −1 for negative coefficient). The legend on the bottom side of the correlogram shows the Spearman correlation coefficients with their corresponding colours. Correlation matrix was made using 'corrplot' package in R (Taiyun and Viliam, 2021).

References

Taiyun, W. and Viliam, S. (2021). R package "corrplot": Visualization of a Correlation Matrix (Version 0.92). Available from https://github.com/taiyun/corrplot.

Table S4. Results of Collinearity Diagnostics for general models examining the effects of the individual variables: sex, age, body condition of three bird species: A) blue tit, B) great tit, C) collared flycatcher, and the year of the study on the status of infection with:
1) *Haemoproteus*; 2) *Plasmodium*; 3) *Trypanosoma*.

A) Blue tit

**Term:**	**AGE**	**SEX**	**CONDITION**	**YEAR**
1) *Haemoproteus*	1.01	1.00	1.03	1.03
2) *Plasmodium*	1.05	1.02	1.12	1.11
3) *Trypanosoma*	1.05	1.00	1.04	1.04

B) Great tit

**Term:**	**AGE**	**SEX**	**CONDITION**	**YEAR**
1) *Haemoproteus*	1.23	1.13	1.14	1.23
2) *Plasmodium*	1.12	1.11	1.01	1.04
3) *Trypanosoma*	1.19	1.12	1.03	1.11

C) Collared flycatcher

**Term:**	**AGE**	**SEX**	**CONDITION**	**YEAR**
1) *Haemoproteus*	1.04	1.02	1.01	1.01
2) *Plasmodium**	* Models were not analyzed due to the too small number of infected individuals (10)			
3) *Trypanosoma*	1.04	1.02	1.02	1.01

Table S5. Results of Collinearity Diagnostics for general models examining the effects of the landscape and population variables: woodland edge, coast edge, NDVI, NDMI, the local density of all bird species, the local density of the same species on the status of infection with: 1) *Haemoproteus*; 2) *Plasmodium*; 3) *Trypanosoma* in three bird species: A) blue tit, B) great tit; C) collared flycatcher.

A) Blue tit

**Term:**	**AllSpecDens**	**OwnSpecDens**	**NDMI**	**NDVI**	**EdgeForestDist**	**CoastEdge**	**Pasture**	**Field**
1) *Haemoproteus*	1.26	1.17	1.74	1.66	1.48	1.65	2.04	1.31
2) *Plasmodium*	1.23	1.20	1.77	1.88	1.47	1.92	2.48	1.14
3) *Trypanosoma*	1.23	1.42	1.89	1.73	1.57	2.20	2.62	1.26

B) Great tit

**Term:**	**AllSpecDens**	**OwnSpecDens**	**NDMI**	**NDVI**	**EdgeForestDist**	**CoastEdge**	**Pasture**	**Field**
1) *Haemoproteus*	1.33	1.41	1.09	1.24	1.69	1.61	1.97	1.35
2) *Plasmodium*	1.34	1.43	1.09	1.24	1.59	1.61	1.87	1.35
3) *Trypanosoma*	1.38	1.44	1.09	1.22	1.52	1.84	2.03	1.30

C) Collared flycatcher

**Term:**	**AllSpecDens**	**OwnSpecDens**	**NDMI**	**NDVI**	**EdgeForestDist**	**CoastEdge**	**Pasture**	**Field**
1) *Haemoproteus*	1.59	1.68	1.13	1.30	1.26	1.47	1.78	1.02
2) *Plasmodium**	* Models were not analyzed due to the too small number of infected individuals (10)							
3) *Trypanosoma*	1.59	1.69	1.15	1.33	1.34	1.43	1.76	1.04

Table S6. AICc-ranked candidate models (within ΔAICc < 4) examining the effects of the individual traits: sex, age, body condition of three bird species: A) blue tit, B) great tit, C) collared flycatcher, and the year of the study on the status of infection with: 1) *Haemoproteus* ; 2) *Plasmodium* ; 3) *Trypanosoma*.

1) *Haemoproteus*

Model	Terms	K	AICc	ΔAICc	Evidence ratio	Log-likelihood	ωAICc	Cumulative AICc
**A) Blue tit**								
1	YEAR	2	75.42	0.00	1.00	-35.62	0.29	0.29
2	CONDITION + YEAR	3	75.84	0.42	1.23	-34.73	0.23	0.52
3	AGE + YEAR	3	76.84	1.42	2.03	-35.23	0.14	0.66
4	SEX + YEAR	3	77.48	2.06	2.80	-35.55	0.10	0.77
5	AGE + CONDITION + YEAR	4	77.49	2.07	2.82	-34.42	0.10	0.87
6	CONDITION + SEX + YEAR	4	77.97	2.55	3.59	-34.66	0.08	0.95
7	AGE + SEX + YEAR	4	78.91	3.49	5.73	-35.13	0.05	1.00
**B) Great tit**								
1	CONDITION + SEX + YEAR	4	301.78	0.00	1.00	-146.81	0.41	0.41
2	AGE + CONDITION + SEX + YEAR	5	302.79	1.01	1.66	-146.28	0.24	0.65
3	AGE + CONDITION + YEAR	4	303.16	1.38	2.00	-147.51	0.20	0.85
4	CONDITION + YEAR	3	303.82	2.04	2.77	-148.86	0.15	1.00
**C) Collared flycatcher**								
1	null	1	343.28	0.00	1.00	-170.63	0.32	0.32
2	SEX	2	344.66	1.37	1.99	-170.30	0.16	0.48
3	YEAR	2	345.31	2.03	2.75	-170.63	0.12	0.60
4	CONDITION	2	345.31	2.03	2.76	-170.63	0.12	0.71
5	AGE	2	345.31	2.03	2.76	-170.63	0.12	0.83

6	AGE + SEX	3	346.69	3.41	5.50	-170.30	0.06	0.88
7	CONDITION + SEX	3	346.70	3.41	5.51	-170.30	0.06	0.94
8	SEX + YEAR	3	346.70	3.42	5.52	-170.30	0.06	1.00

2) ***Plasmodium***

Model	Terms	K	AICc	ΔAICc	Evidence ratio	Log-likelihood	ωAICc	Cumulative AICc
**A) Blue tit**								
1	CONDITION + YEAR	3	72.24	0.00	1.00	-32.93	0.41	0.41
2	CONDITION + SEX + YEAR	4	73.47	1.23	1.85	-32.41	0.22	0.62
3	AGE + CONDITION + YEAR	4	73.61	1.37	1.98	-32.48	0.20	0.83
4	AGE + CONDITION + SEX + YEAR	5	74.78	2.54	3.56	-31.90	0.11	0.94
5	CONDITION	2	76.18	3.94	7.15	-36.00	0.06	1.00
**B) Great tit**								
1	AGE + CONDITION + YEAR	4	320.92	0.00	1.00	-156.39	0.19	0.19
2	CONDITION + SEX + YEAR	4	321.35	0.43	1.24	-156.60	0.15	0.34
3	CONDITION + YEAR	3	321.46	0.54	1.31	-157.69	0.14	0.48
4	AGE + YEAR	3	321.65	0.73	1.44	-157.78	0.13	0.61
5	AGE + CONDITION + SEX + YEAR	5	321.95	1.03	1.67	-155.87	0.11	0.72
6	SEX + YEAR	3	322.14	1.22	1.84	-158.03	0.10	0.83
7	YEAR	2	322.27	1.34	1.96	-159.11	0.10	0.92
8	AGE + SEX + YEAR	4	322.68	1.76	2.41	-157.27	0.08	1.00
**C) Collared flycatcher***								

*** Models were not analyzed due to the too small number of infected individuals (10)**

3) *Trypanosoma*

Model	Terms	K	AICc	ΔAICc	Evidence ratio	Log-likelihood	ωAICc	Cumulative AICc
**A) Blue tit**								
1	CONDITION + YEAR	3	64.69	0.00	1.00	-29.15	0.13	0.13
2	YEAR	2	64.75	0.06	1.03	-30.28	0.12	0.25
3	null	1	65.05	0.36	1.20	-31.49	0.11	0.35
4	CONDITION	2	65.05	0.36	1.20	-30.43	0.10	0.46
5	AGE + CONDITION + YEAR	4	65.24	0.55	1.32	-28.30	0.10	0.55
6	AGE + YEAR	3	65.62	0.93	1.59	-29.62	0.08	0.63
7	AGE + CONDITION	3	66.04	1.35	1.96	-29.83	0.06	0.70
8	AGE	2	66.32	1.63	2.26	-31.06	0.06	0.75
9	SEX + YEAR	3	66.92	2.23	3.05	-30.27	0.04	0.79
10	CONDITION + SEX + YEAR	4	66.94	2.25	3.08	-29.15	0.04	0.83
11	SEX	2	67.14	2.45	3.41	-31.48	0.04	0.87
12	CONDITION + SEX	3	67.22	2.53	3.54	-30.42	0.04	0.91
13	AGE + CONDITION + SEX + YEAR	5	67.57	2.88	4.23	-28.29	0.03	0.94
14	AGE + SEX + YEAR	4	67.87	3.18	4.90	-29.61	0.03	0.96
15	AGE + CONDITION + SEX	4	68.30	3.61	6.08	-29.83	0.02	0.98
16	AGE + SEX	3	68.50	3.81	6.72	-31.06	0.02	1.00
**B) Great tit**								
1	CONDITION	2	178.28	0.00	1.00	-87.12	0.12	0.12
2	null	1	178.60	0.32	1.18	-88.29	0.10	0.22
3	CONDITION + YEAR	3	179.03	0.75	1.46	-86.47	0.08	0.31
4	CONDITION + SEX	3	179.08	0.80	1.50	-86.50	0.08	0.39

5	AGE + CONDITION + SEX	4	179.09	0.81	1.50	-85.47	0.08	0.47
6	AGE + CONDITION	3	179.27	0.99	1.64	-86.59	0.07	0.54
7	SEX	2	179.39	1.11	1.74	-87.67	0.07	0.61
8	AGE + SEX	3	179.58	1.30	1.92	-86.75	0.06	0.67
9	AGE	2	179.74	1.46	2.08	-87.85	0.06	0.73
10	CONDITION + SEX + YEAR	4	179.91	1.63	2.26	-85.88	0.05	0.78
11	YEAR	2	179.96	1.69	2.32	-87.96	0.05	0.84
12	AGE + CONDITION + YEAR	4	180.48	2.20	3.01	-86.17	0.04	0.88
13	AGE + CONDITION + SEX + YEAR	5	180.60	2.32	3.19	-85.19	0.04	0.91
14	SEX + YEAR	3	180.78	2.50	3.49	-87.34	0.03	0.95
15	AGE + YEAR	3	181.39	3.11	4.74	-87.65	0.03	0.97
16	AGE + SEX + YEAR	4	181.40	3.12	4.76	-86.62	0.03	1.00
**C) Collared flycatcher**								
1	YEAR	2	300.76	0.00	1.00	-148.36	0.26	0.26
2	AGE + YEAR	3	301.51	0.75	1.45	-147.71	0.18	0.45
3	CONDITION + YEAR	3	302.69	1.93	2.62	-148.30	0.10	0.55
4	SEX + YEAR	3	302.73	1.96	2.67	-148.32	0.10	0.65
5	null	1	302.85	2.09	2.84	-150.42	0.09	0.74
6	AGE	2	303.14	2.38	3.29	-149.55	0.08	0.82
7	AGE + CONDITION + YEAR	4	303.35	2.59	3.65	-147.60	0.07	0.89
8	AGE + SEX + YEAR	4	303.37	2.60	3.68	-147.61	0.07	0.96
9	CONDITION + SEX + YEAR	4	304.67	3.91	7.05	-148.26	0.04	1.00

Table S7. AICc-ranked candidate models (within ΔAICc < 4) examining the effects of the landscape and population variables: the local density of all bird species, the local density of the same species, woodland edge, coast edge, NDVI, NDMI, distance to the field, distance to the pasture on the status of infection with: 1) *Haemoproteus* ; 2) *Plasmodium* ; 3) *Trypanosoma* in three bird species: A) blue tit, B) great tit, C) collared flycatcher.

1) *Haemoproteus*

Model	Terms	K	AICc	ΔAICc	Evidence ratio	Log-likelihood	ωAICc	Cumulative AICc
**A) Blue tit**								
1	NDMI	2	88.09	0.00	1.00	-41.95	0.14	0.14
2	NDMI+Pasture	3	88.86	0.78	1.47	-41.24	0.10	0.24
3	AllSpecDens+NDMI	3	89.55	1.46	2.07	-41.58	0.07	0.31
4	NDMI+NDVI	3	89.90	1.82	2.48	-41.76	0.06	0.37
5	OwnSpecDens+NDMI	3	89.93	1.85	2.52	-41.78	0.06	0.42
6	NDMI+Field	3	89.98	1.90	2.58	-41.80	0.06	0.48
7	ForestEdge+NDMI	3	90.26	2.17	2.97	-41.94	0.05	0.53
8	AllSpecDens+NDMI+Pasture	4	90.27	2.19	2.98	-40.81	0.05	0.57
9	CoastEdge+NDMI	3	90.28	2.19	2.99	-41.95	0.05	0.62
10	NDMI+Field+Pasture	4	90.73	2.64	3.74	-41.04	0.04	0.66
11	OwnSpecDens+NDMI+Pasture	4	90.85	2.76	3.98	-41.10	0.04	0.70
12	CoastEdge+NDMI+Pasture	4	90.90	2.81	4.08	-41.13	0.03	0.73
13	ForestEdge+NDMI+Pasture	4	91.01	2.93	4.32	-41.18	0.03	0.76
14	NDMI+NDVI+Pasture	4	91.12	3.04	4.57	-41.24	0.03	0.79
15	AllSpecDens+NDMI+Field	4	91.34	3.25	5.09	-41.35	0.03	0.82
16	AllSpecDens+NDMI+NDVI	4	91.57	3.48	5.70	-41.46	0.03	0.85

17	AllSpecDens+OwnSpecDens+NDMI	4	91.58	3.49	5.73	-41.47	0.02	0.87
18	AllSpecDens+CoastEdge+NDMI	4	91.81	3.72	6.43	-41.58	0.02	0.89
19	AllSpecDens+ForestEdge+NDMI	4	91.81	3.72	6.43	-41.58	0.02	0.92
20	OwnSpecDens+NDMI+NDVI	4	91.82	3.73	6.47	-41.59	0.02	0.94
21	NDMI+NDVI+Field	4	91.91	3.82	6.75	-41.63	0.02	0.96
22	AllSpecDens+NDMI+Field+Pasture	5	92.00	3.91	7.07	-40.51	0.02	0.98
23	OwnSpecDens+NDMI+Field	4	92.01	3.92	7.10	-41.68	0.02	1.00
**B) Great tit**								
1	OwnSpecDens	2	337.50	0.00	1.00	-166.73	0.04	0.04
2	OwnSpecDens+NDMI	3	337.59	0.09	1.05	-165.75	0.04	0.07
3	OwnSpecDens+ForestEdge+Pasture	4	338.01	0.51	1.29	-164.93	0.03	0.10
4	OwnSpecDens+NDVI	3	338.16	0.66	1.39	-166.04	0.03	0.13
5	OwnSpecDens+ForestEdge+NDMI+Pasture	5	338.19	0.69	1.41	-163.98	0.03	0.16
6	OwnSpecDens+NDMI+NDVI	4	338.24	0.73	1.44	-165.04	0.03	0.18
7	AllSpecDens+OwnSpecDens	3	338.36	0.86	1.54	-166.14	0.02	0.21
8	OwnSpecDens+NDMI+Field	4	338.62	1.12	1.75	-165.24	0.02	0.23
9	OwnSpecDens+ForestEdge+NDMI	4	338.64	1.13	1.76	-165.24	0.02	0.25
10	OwnSpecDens+ForestEdge	3	338.67	1.16	1.79	-166.29	0.02	0.27
11	OwnSpecDens+Pasture	3	338.67	1.17	1.80	-166.29	0.02	0.29
12	AllSpecDens+OwnSpecDens+NDMI	4	338.73	1.23	1.85	-165.29	0.02	0.31
13	AllSpecDens+OwnSpecDens+ForestEdge+Pasture	5	338.89	1.38	2.00	-164.33	0.02	0.33
14	OwnSpecDens+Field	3	338.95	1.45	2.06	-166.43	0.02	0.35
15	OwnSpecDens+NDMI+Pasture	4	338.97	1.47	2.08	-165.41	0.02	0.37
16	AllSpecDens+OwnSpecDens+NDVI	4	339.34	1.84	2.51	-165.60	0.02	0.38
17	AllSpecDens+OwnSpecDens+ForestEdge+NDMI+Pasture	6	339.35	1.84	2.51	-163.52	0.02	0.40

18	OwnSpecDens+CoastEdge+NDMI	4	339.38	1.87	2.55	-165.61	0.01	0.41
19	OwnSpecDens+CoastEdge	3	339.44	1.94	2.64	-166.68	0.01	0.43
20	OwnSpecDens+ForestEdge+NDMI+NDVI	5	339.45	1.95	2.65	-164.62	0.01	0.44
21	OwnSpecDens+ForestEdge+NDVI	4	339.49	1.99	2.70	-165.67	0.01	0.46
22	AllSpecDens+OwnSpecDens+Pasture	4	339.53	2.02	2.75	-165.69	0.01	0.47
23	AllSpecDens+OwnSpecDens+ForestEdge	4	339.55	2.05	2.79	-165.70	0.01	0.48
24	OwnSpecDens+ForestEdge+NDVI+Pasture	5	339.65	2.15	2.93	-164.72	0.01	0.50
25	AllSpecDens+OwnSpecDens+NDMI+NDVI	5	339.68	2.17	2.96	-164.73	0.01	0.51
26	OwnSpecDens+NDVI+Pasture	4	339.73	2.23	3.05	-165.79	0.01	0.52
27	OwnSpecDens+ForestEdge+NDMI+NDVI+Pasture	6	339.79	2.28	3.13	-163.74	0.01	0.53
28	AllSpecDens+OwnSpecDens+ForestEdge+NDMI	5	339.81	2.30	3.16	-164.79	0.01	0.55
29	AllSpecDens+OwnSpecDens+NDMI+Field	5	339.84	2.34	3.22	-164.81	0.01	0.56
30	AllSpecDens+OwnSpecDens+Field	4	339.86	2.36	3.25	-165.86	0.01	0.57
31	OwnSpecDens+NDMI+NDVI+Field	5	339.88	2.38	3.29	-164.83	0.01	0.58
32	OwnSpecDens+NDMI+NDVI+Pasture	5	339.95	2.45	3.41	-164.87	0.01	0.59
33	OwnSpecDens+ForestEdge+CoastEdge+NDMI	5	339.97	2.46	3.42	-164.87	0.01	0.60
34	OwnSpecDens+ForestEdge+Field+Pasture	5	340.03	2.53	3.54	-164.90	0.01	0.61
35	OwnSpecDens+NDMI+Field+Pasture	5	340.05	2.55	3.57	-164.91	0.01	0.62
36	OwnSpecDens+NDVI+Field	4	340.05	2.55	3.57	-165.95	0.01	0.64
37	OwnSpecDens+ForestEdge+CoastEdge+Pasture	5	340.07	2.57	3.61	-164.92	0.01	0.65
38	OwnSpecDens+ForestEdge+NDMI+Field+Pasture	6	340.07	2.57	3.61	-163.88	0.01	0.66
39	AllSpecDens+OwnSpecDens+NDMI+Pasture	5	340.10	2.59	3.66	-164.94	0.01	0.67
40	OwnSpecDens+ForestEdge+NDMI+Field	5	340.11	2.60	3.68	-164.94	0.01	0.68
41	OwnSpecDens+Field+Pasture	4	340.14	2.64	3.74	-166.00	0.01	0.69
42	OwnSpecDens+CoastEdge+NDVI	4	340.21	2.71	3.88	-166.03	0.01	0.70

43	OwnSpecDens+CoastEdge+NDMI+NDVI	5	340.23	2.73	3.91	-165.00	0.01	0.71
44	OwnSpecDens+ForestEdge+CoastEdge+NDMI+Pasture	6	340.26	2.75	3.96	-163.97	0.01	0.72
45	NDMI+NDVI	3	340.27	2.77	4.00	-167.09	0.01	0.73
46	NDMI	2	340.32	2.82	4.09	-168.14	0.01	0.73
47	OwnSpecDens+ForestEdge+CoastEdge	4	340.35	2.85	4.15	-166.10	0.01	0.74
48	AllSpecDens+OwnSpecDens+CoastEdge	4	340.39	2.89	4.24	-166.12	0.01	0.75
49	OwnSpecDens+ForestEdge+Field	4	340.43	2.93	4.32	-166.14	0.01	0.76
50	NDVI	2	340.44	2.94	4.35	-168.20	0.01	0.77
51	null	1	340.49	2.99	4.46	-169.24	0.01	0.78
52	OwnSpecDens+CoastEdge+NDMI+Field	5	340.58	3.08	4.66	-165.18	0.01	0.79
53	ForestEdge+NDMI+Pasture	4	340.63	3.13	4.78	-166.24	0.01	0.79
54	AllSpecDens+OwnSpecDens+CoastEdge+NDMI	5	340.67	3.16	4.86	-165.22	0.01	0.80
55	ForestEdge+Pasture	3	340.67	3.16	4.86	-167.29	0.01	0.81
56	AllSpecDens+OwnSpecDens+ForestEdge+NDVI	5	340.68	3.18	4.90	-165.23	0.01	0.82
57	OwnSpecDens+CoastEdge+Pasture	4	340.71	3.21	4.97	-166.28	0.01	0.83
58	AllSpecDens+OwnSpecDens+ForestEdge+NDVI+Pasture	6	340.72	3.21	4.99	-164.20	0.01	0.83
59	Pasture	2	340.73	3.22	5.01	-168.34	0.01	0.84
60	NDMI+Pasture	3	340.86	3.36	5.36	-167.39	0.01	0.85
61	AllSpecDens+OwnSpecDens+ForestEdge+CoastEdge+Pasture	6	340.86	3.36	5.36	-164.27	0.01	0.85
62	AllSpecDens+OwnSpecDens+NDVI+Pasture	5	340.87	3.37	5.39	-165.32	0.01	0.86
63	AllSpecDens+OwnSpecDens+ForestEdge+NDMI+NDVI	6	340.91	3.41	5.49	-164.30	0.01	0.87
64	AllSpecDens+OwnSpecDens+ForestEdge+Field+Pasture	6	340.93	3.43	5.55	-164.31	0.01	0.88
65	OwnSpecDens+CoastEdge+Field	4	340.98	3.48	5.69	-166.42	0.01	0.88
66	OwnSpecDens+CoastEdge+NDMI+Pasture	5	341.03	3.53	5.84	-165.40	0.01	0.89
67	AllSpecDens+OwnSpecDens+Field+Pasture	5	341.05	3.55	5.90	-165.42	0.01	0.90

68	AllSpecDens+OwnSpecDens+ForestEdge+NDMI+NDVI+Pasture	7	341.14	3.63	6.15	-163.36	0.01	0.90
69	OwnSpecDens+ForestEdge+CoastEdge+NDMI+NDVI	6	341.18	3.68	6.29	-164.43	0.01	0.91
70	AllSpecDens+OwnSpecDens+NDVI+Field	5	341.23	3.72	6.43	-165.50	0.01	0.91
71	AllSpecDens+OwnSpecDens+NDMI+Field+Pasture	6	341.27	3.76	6.56	-164.48	0.01	0.92
72	AllSpecDens+OwnSpecDens+ForestEdge+NDMI+Field+Pasture	7	341.27	3.77	6.58	-163.43	0.01	0.92
73	AllSpecDens+OwnSpecDens+NDMI+NDVI+Field	6	341.32	3.82	6.75	-164.50	0.01	0.93
74	AllSpecDens+OwnSpecDens+ForestEdge+NDMI+Field	6	341.34	3.84	6.81	-164.51	0.01	0.94
75	AllSpecDens+OwnSpecDens+ForestEdge+Field	5	341.35	3.85	6.85	-165.56	0.01	0.94
76	AllSpecDens+OwnSpecDens+NDMI+NDVI+Pasture	6	341.36	3.86	6.89	-164.52	0.01	0.95
77	OwnSpecDens+ForestEdge+NDMI+NDVI+Field	6	341.37	3.86	6.90	-164.53	0.01	0.95
78	AllSpecDens+OwnSpecDens+ForestEdge+CoastEdge+NDMI	6	341.39	3.89	6.99	-164.54	0.01	0.96
79	CoastEdge+NDMI	3	341.39	3.89	6.99	-167.65	0.01	0.96
80	AllSpecDens+OwnSpecDens+CoastEdge+NDVI	5	341.42	3.91	7.08	-165.60	0.01	0.97
81	NDVI+Pasture	3	341.42	3.92	7.08	-167.67	0.01	0.97
82	AllSpecDens+OwnSpecDens+ForestEdge+CoastEdge	5	341.43	3.92	7.11	-165.60	0.01	0.98
83	OwnSpecDens+ForestEdge+CoastEdge+NDVI	5	341.44	3.93	7.15	-165.61	0.01	0.98
84	AllSpecDens+OwnSpecDens+ForestEdge+CoastEdge+NDMI+Pasture	7	341.45	3.95	7.19	-163.51	0.01	0.99
85	AllSpecDens+OwnSpecDens+CoastEdge+Pasture	5	341.47	3.97	7.26	-165.62	0.01	0.99
86	NDMI+NDVI+Pasture	4	341.47	3.97	7.27	-166.66	0.01	1.00
**C) Collared flycatcher**								
1	AllSpecDens+ForestEdge+CoastEdge+Field	5	335.27	0.00	1.00	-162.52	0.05	0.05
2	AllSpecDens+ForestEdge+Field	4	335.51	0.23	1.12	-163.68	0.05	0.10
3	AllSpecDens+ForestEdge+CoastEdge+NDVI+Field	6	335.59	0.32	1.17	-161.64	0.05	0.15
4	AllSpecDens+ForestEdge+CoastEdge+Field+Pasture	6	336.03	0.75	1.46	-161.85	0.04	0.18
5	AllSpecDens+OwnSpecDens+ForestEdge+CoastEdge+Field	6	336.13	0.86	1.54	-161.90	0.03	0.22

6	AllSpecDens+CoastEdge+Field	4	336.14	0.86	1.54	-163.99	0.03	0.25
7	AllSpecDens+OwnSpecDens+CoastEdge+Field	5	336.54	1.27	1.89	-163.16	0.03	0.28
8	AllSpecDens+ForestEdge+NDVI+Field	5	336.59	1.32	1.93	-163.18	0.03	0.31
9	AllSpecDens+CoastEdge+NDVI+Field	5	336.64	1.36	1.98	-163.20	0.03	0.33
10	AllSpecDens+ForestEdge	3	336.83	1.55	2.18	-165.37	0.02	0.36
11	AllSpecDens+OwnSpecDens+ForestEdge+Field	5	336.87	1.59	2.22	-163.32	0.02	0.38
12	AllSpecDens+ForestEdge+CoastEdge+NDVI	5	336.90	1.63	2.25	-163.34	0.02	0.41
13	AllSpecDens+OwnSpecDens+ForestEdge+CoastEdge+NDVI+Field	7	337.02	1.75	2.39	-161.29	0.02	0.43
14	AllSpecDens+ForestEdge+CoastEdge	4	337.02	1.75	2.40	-164.43	0.02	0.45
15	AllSpecDens+ForestEdge+CoastEdge+NDVI+Field+Pasture	7	337.06	1.78	2.44	-161.31	0.02	0.47
16	AllSpecDens+OwnSpecDens+ForestEdge+CoastEdge+Field+Pasture	7	337.19	1.91	2.60	-161.38	0.02	0.49
17	AllSpecDens+ForestEdge+CoastEdge+NDMI+Field	6	337.35	2.08	2.83	-162.52	0.02	0.51
18	AllSpecDens+ForestEdge+NDVI	4	337.49	2.21	3.02	-164.67	0.02	0.53
19	AllSpecDens+ForestEdge+NDMI+Field	5	337.49	2.22	3.03	-163.63	0.02	0.55
20	AllSpecDens+ForestEdge+Field+Pasture	5	337.51	2.23	3.05	-163.64	0.02	0.56
21	AllSpecDens+OwnSpecDens+CoastEdge+NDVI+Field	6	337.65	2.38	3.28	-162.66	0.02	0.58
22	AllSpecDens+ForestEdge+CoastEdge+NDMI+NDVI+Field	7	337.66	2.38	3.29	-161.61	0.02	0.60
23	AllSpecDens+OwnSpecDens+ForestEdge+CoastEdge	5	337.72	2.45	3.40	-163.75	0.02	0.61
24	ForestEdge+CoastEdge+Field+Pasture	5	337.73	2.46	3.42	-163.75	0.02	0.63
25	ForestEdge+CoastEdge+Field	4	337.86	2.59	3.64	-164.85	0.01	0.64
26	AllSpecDens+ForestEdge+CoastEdge+Pasture	5	337.87	2.60	3.66	-163.82	0.01	0.66
27	AllSpecDens+CoastEdge+Field+Pasture	5	337.92	2.65	3.76	-163.85	0.01	0.67
28	AllSpecDens+OwnSpecDens+ForestEdge	4	338.02	2.75	3.95	-164.94	0.01	0.68
29	CoastEdge+Field	3	338.06	2.78	4.02	-165.98	0.01	0.70
30	AllSpecDens+ForestEdge+CoastEdge+NDMI+Field+Pasture	7	338.13	2.86	4.18	-161.85	0.01	0.71

31	AllSpecDens+OwnSpecDens+ForestEdge+CoastEdge+NDMI+Field	7	338.17	2.90	4.26	-161.87	0.01	0.72
32	AllSpecDens+CoastEdge+NDMI+Field	5	338.22	2.94	4.35	-163.99	0.01	0.74
33	AllSpecDens+OwnSpecDens+ForestEdge+CoastEdge+NDVI	6	338.27	3.00	4.48	-162.97	0.01	0.75
34	AllSpecDens+OwnSpecDens+ForestEdge+NDVI+Field	6	338.31	3.04	4.56	-162.99	0.01	0.76
35	ForestEdge+CoastEdge+NDVI+Field	5	338.33	3.06	4.62	-164.05	0.01	0.77
36	ForestEdge+Field	3	338.33	3.06	4.62	-166.12	0.01	0.78
37	AllSpecDens+OwnSpecDens+CoastEdge+Field+Pasture	6	338.46	3.18	4.91	-163.07	0.01	0.79
38	AllSpecDens+ForestEdge+CoastEdge+NDVI+Pasture	6	338.53	3.25	5.09	-163.10	0.01	0.80
39	AllSpecDens+OwnSpecDens+ForestEdge+CoastEdge+NDVI+Field+Pasture	8	338.58	3.31	5.23	-161.01	0.01	0.81
40	AllSpecDens+OwnSpecDens+CoastEdge+NDMI+Field	6	338.60	3.32	5.27	-163.14	0.01	0.82
41	AllSpecDens+Field	3	338.60	3.33	5.28	-166.26	0.01	0.83
42	AllSpecDens+CoastEdge+NDMI+NDVI+Field	6	338.64	3.37	5.39	-163.16	0.01	0.84
43	AllSpecDens+ForestEdge+NDMI+NDVI+Field	6	338.68	3.40	5.49	-163.18	0.01	0.85
44	AllSpecDens+ForestEdge+NDVI+Field+Pasture	6	338.68	3.41	5.50	-163.18	0.01	0.86
45	CoastEdge+NDVI+Field	4	338.68	3.41	5.50	-165.27	0.01	0.87
46	AllSpecDens+CoastEdge+NDVI+Field+Pasture	6	338.69	3.42	5.52	-163.18	0.01	0.88
47	AllSpecDens+ForestEdge+Pasture	4	338.77	3.50	5.76	-165.31	0.01	0.89
48	AllSpecDens+OwnSpecDens+ForestEdge+NDMI+Field	6	338.78	3.50	5.77	-163.23	0.01	0.90
49	AllSpecDens+ForestEdge+NDMI	4	338.80	3.53	5.83	-165.32	0.01	0.91
50	AllSpecDens+OwnSpecDens+ForestEdge+CoastEdge+Pasture	6	338.87	3.59	6.03	-163.27	0.01	0.92
51	AllSpecDens+ForestEdge+CoastEdge+NDMI+NDVI	6	338.94	3.66	6.24	-163.31	0.01	0.93
52	AllSpecDens+CoastEdge	3	338.95	3.67	6.27	-166.43	0.01	0.94
53	AllSpecDens+OwnSpecDens+ForestEdge+Field+Pasture	6	338.95	3.67	6.27	-163.31	0.01	0.94
54	AllSpecDens+CoastEdge+NDVI	4	338.95	3.67	6.27	-165.40	0.01	0.95
55	ForestEdge+CoastEdge+NDVI+Field+Pasture	6	339.04	3.77	6.59	-163.36	0.01	0.96

56	AllSpecDens+OwnSpecDens+CoastEdge	4	339.06	3.79	6.65	-165.46	0.01	0.97
57	AllSpecDens+ForestEdge+CoastEdge+NDMI	5	339.08	3.81	6.71	-164.43	0.01	0.98
58	AllSpecDens+ForestEdge+CoastEdge+NDMI+NDVI+Field+Pasture	8	339.12	3.85	6.84	-161.28	0.01	0.98
59	AllSpecDens+OwnSpecDens+ForestEdge+CoastEdge+NDMI+NDVI+Field	8	339.14	3.87	6.92	-161.29	0.01	0.99
60	AllSpecDens+OwnSpecDens+ForestEdge+NDVI	5	339.14	3.87	6.92	-164.46	0.01	1.00

2) *Plasmodium*

Model	Terms	K	AICc	ΔAICc	Evidence ratio	Log-likelihood	ωAICc	Cumulative AICc
**A) Blue tit**								
1	ForestEdge+CoastEdge+NDVI+Pasture	5	76.57	0.00	1.00	-32.79	0.05	0.05
2	ForestEdge+Field+Pasture	4	76.85	0.27	1.15	-34.10	0.04	0.09
3	ForestEdge+NDVI+Field+Pasture	5	76.99	0.42	1.23	-33.00	0.04	0.14
4	AllSpecDens+ForestEdge+CoastEdge+NDVI+Pasture	6	77.11	0.54	1.31	-31.85	0.04	0.17
5	ForestEdge+CoastEdge+NDVI+Field+Pasture	6	77.31	0.74	1.45	-31.96	0.04	0.21
6	ForestEdge+CoastEdge+NDMI+Pasture	5	77.39	0.82	1.51	-33.20	0.03	0.24
7	ForestEdge+CoastEdge+Pasture	4	77.39	0.82	1.51	-34.37	0.03	0.28
8	ForestEdge+CoastEdge+Field+Pasture	5	77.82	1.25	1.87	-33.42	0.03	0.30
9	ForestEdge+NDVI+Pasture	4	78.05	1.48	2.10	-34.70	0.02	0.33
10	CoastEdge+NDMI+Pasture	4	78.07	1.50	2.12	-34.71	0.02	0.35
11	ForestEdge+Pasture	3	78.09	1.52	2.14	-35.86	0.02	0.38
12	ForestEdge+CoastEdge+NDMI+NDVI+Pasture	6	78.22	1.64	2.27	-32.41	0.02	0.40
13	CoastEdge+Pasture	3	78.37	1.80	2.46	-36.00	0.02	0.42
14	AllSpecDens+ForestEdge+CoastEdge+Pasture	5	78.39	1.82	2.49	-33.71	0.02	0.44
15	AllSpecDens+ForestEdge+CoastEdge+NDVI+Field+Pasture	7	78.44	1.87	2.54	-31.27	0.02	0.46
16	ForestEdge+CoastEdge+NDMI+Field+Pasture	6	78.49	1.91	2.60	-32.54	0.02	0.48

17	ForestEdge+Field	3	78.58	2.01	2.73	-36.10	0.02	0.50
18	OwnSpecDens+ForestEdge+Field+Pasture	5	78.64	2.06	2.81	-33.83	0.02	0.52
19	OwnSpecDens+ForestEdge+NDVI+Field+Pasture	6	78.64	2.07	2.81	-32.62	0.02	0.53
20	AllSpecDens+ForestEdge+NDVI+Field+Pasture	6	78.68	2.10	2.86	-32.64	0.02	0.55
21	AllSpecDens+ForestEdge+Field+Pasture	5	78.71	2.14	2.91	-33.86	0.02	0.57
22	ForestEdge+NDMI+Field+Pasture	5	78.71	2.14	2.91	-33.86	0.02	0.59
23	AllSpecDens+ForestEdge+CoastEdge+NDMI+Pasture	6	78.79	2.21	3.03	-32.69	0.02	0.60
24	OwnSpecDens+ForestEdge+CoastEdge+NDVI+Pasture	6	78.81	2.23	3.06	-32.70	0.02	0.62
25	Field	2	79.03	2.45	3.41	-37.42	0.01	0.63
26	CoastEdge+NDVI+Pasture	4	79.08	2.51	3.51	-35.22	0.01	0.65
27	OwnSpecDens+ForestEdge+CoastEdge+NDMI+Pasture	6	79.12	2.54	3.57	-32.86	0.01	0.66
28	CoastEdge+Field+Pasture	4	79.14	2.57	3.62	-35.25	0.01	0.68
29	AllSpecDens+ForestEdge+NDVI+Pasture	5	79.23	2.65	3.77	-34.12	0.01	0.69
30	AllSpecDens+ForestEdge+CoastEdge+NDMI+NDVI+Pasture	7	79.26	2.69	3.84	-31.68	0.01	0.70
31	Field+Pasture	3	79.27	2.70	3.85	-36.44	0.01	0.72
32	OwnSpecDens+ForestEdge+CoastEdge+NDVI+Field+Pasture	7	79.29	2.72	3.89	-31.70	0.01	0.73
33	AllSpecDens+ForestEdge+CoastEdge+Field+Pasture	6	79.33	2.76	3.98	-32.97	0.01	0.74
34	ForestEdge+CoastEdge+NDMI+NDVI+Field+Pasture	7	79.35	2.77	4.00	-31.72	0.01	0.76
35	CoastEdge+NDMI+Field+Pasture	5	79.37	2.80	4.05	-34.19	0.01	0.77
36	ForestEdge+NDMI+NDVI+Field+Pasture	6	79.40	2.83	4.11	-33.00	0.01	0.78
37	AllSpecDens+OwnSpecDens+ForestEdge+CoastEdge+NDVI+Pasture	7	79.42	2.85	4.16	-31.76	0.01	0.79
38	AllSpecDens+ForestEdge+Pasture	4	79.56	2.99	4.45	-35.46	0.01	0.80
39	OwnSpecDens+ForestEdge+CoastEdge+Pasture	5	79.66	3.09	4.68	-34.34	0.01	0.81
40	ForestEdge+NDMI+Pasture	4	79.74	3.17	4.88	-35.55	0.01	0.82
41	CoastEdge+NDMI+NDVI+Pasture	5	79.77	3.19	4.94	-34.39	0.01	0.84

42	OwnSpecDens+ForestEdge+CoastEdge+Field+Pasture	6	79.89	3.32	5.25	-33.24	0.01	0.84
43	OwnSpecDens+ForestEdge+CoastEdge+NDMI+Field+Pasture	7	79.93	3.36	5.36	-32.02	0.01	0.85
44	null	1	80.04	3.47	5.66	-38.99	0.01	0.86
45	OwnSpecDens+ForestEdge+NDVI+Pasture	5	80.07	3.50	5.76	-34.55	0.01	0.87
46	AllSpecDens+CoastEdge+Pasture	4	80.08	3.51	5.78	-35.72	0.01	0.88
47	ForestEdge	2	80.11	3.54	5.86	-37.96	0.01	0.89
48	OwnSpecDens+ForestEdge+NDMI+Field+Pasture	6	80.15	3.58	5.98	-33.37	0.01	0.90
49	OwnSpecDens+ForestEdge+CoastEdge+NDMI+NDVI+Pasture	7	80.16	3.59	6.02	-32.13	0.01	0.91
50	OwnSpecDens+CoastEdge+NDMI+Pasture	5	80.18	3.61	6.08	-34.60	0.01	0.91
51	AllSpecDens+CoastEdge+NDMI+Pasture	5	80.18	3.61	6.08	-34.60	0.01	0.92
52	OwnSpecDens+ForestEdge+Pasture	4	80.19	3.62	6.11	-35.77	0.01	0.93
53	CoastEdge+NDVI+Field+Pasture	5	80.23	3.65	6.22	-34.62	0.01	0.94
54	AllSpecDens+ForestEdge+CoastEdge+NDMI+Field+Pasture	7	80.24	3.67	6.25	-32.17	0.01	0.95
55	Pasture	2	80.24	3.67	6.27	-38.03	0.01	0.96
56	OwnSpecDens+ForestEdge+Field	4	80.32	3.75	6.52	-35.84	0.01	0.96
57	ForestEdge+NDMI+NDVI+Pasture	5	80.36	3.79	6.65	-34.69	0.01	0.97
58	ForestEdge+NDMI+Field	4	80.42	3.85	6.87	-35.89	0.01	0.98
59	CoastEdge+Field	3	80.47	3.90	7.03	-37.05	0.01	0.99
60	AllSpecDens+ForestEdge+Field	4	80.50	3.93	7.14	-35.93	0.01	0.99
61	AllSpecDens+OwnSpecDens+ForestEdge+NDVI+Field+Pasture	7	80.51	3.94	7.17	-32.31	0.01	1.00
**B) Great tit**								
1	OwnSpecDens+NDMI+Pasture	4	372.89	0.00	1.00	-182.37	0.10	0.10
2	OwnSpecDens+CoastEdge+NDMI	4	373.81	0.92	1.58	-182.83	0.06	0.16
3	OwnSpecDens+NDMI	3	373.92	1.03	1.67	-183.92	0.06	0.21
4	OwnSpecDens+CoastEdge	3	374.27	1.38	2.00	-184.09	0.05	0.26

5	OwnSpecDens+CoastEdge+NDMI+Pasture	5	374.52	1.63	2.26	-182.15	0.04	0.31
6	OwnSpecDens+Pasture	3	374.63	1.74	2.38	-184.27	0.04	0.35
7	OwnSpecDens+ForestEdge+NDMI+Pasture	5	374.67	1.78	2.43	-182.22	0.04	0.39
8	AllSpecDens+OwnSpecDens+NDMI+Pasture	5	374.69	1.81	2.47	-182.24	0.04	0.42
9	OwnSpecDens+NDMI+NDVI+Pasture	5	374.86	1.97	2.68	-182.32	0.04	0.46
10	OwnSpecDens+NDMI+Field+Pasture	5	374.93	2.04	2.78	-182.35	0.03	0.49
11	OwnSpecDens	2	375.19	2.30	3.16	-185.57	0.03	0.53
12	OwnSpecDens+CoastEdge+Pasture	4	375.55	2.67	3.79	-183.70	0.03	0.55
13	OwnSpecDens+CoastEdge+NDMI+Field	5	375.68	2.79	4.04	-182.73	0.02	0.57
14	AllSpecDens+OwnSpecDens+NDMI	4	375.73	2.84	4.13	-183.79	0.02	0.60
15	AllSpecDens+OwnSpecDens+CoastEdge+NDMI	5	375.81	2.92	4.31	-182.79	0.02	0.62
16	OwnSpecDens+ForestEdge+NDMI	4	375.82	2.93	4.33	-183.84	0.02	0.64
17	OwnSpecDens+CoastEdge+NDMI+NDVI	5	375.82	2.93	4.33	-182.80	0.02	0.66
18	OwnSpecDens+ForestEdge+CoastEdge+NDMI	5	375.88	2.99	4.47	-182.83	0.02	0.69
19	OwnSpecDens+NDMI+Field	4	375.96	3.07	4.65	-183.91	0.02	0.71
20	OwnSpecDens+NDMI+NDVI	4	375.97	3.09	4.68	-183.91	0.02	0.73
21	OwnSpecDens+CoastEdge+NDVI	4	376.26	3.37	5.39	-184.06	0.02	0.75
22	OwnSpecDens+CoastEdge+Field	4	376.28	3.39	5.45	-184.07	0.02	0.76
23	OwnSpecDens+ForestEdge+CoastEdge+NDMI+Pasture	6	376.29	3.40	5.47	-181.99	0.02	0.78
24	OwnSpecDens+ForestEdge+CoastEdge	4	376.31	3.42	5.53	-184.08	0.02	0.80
25	AllSpecDens+OwnSpecDens+CoastEdge	4	376.32	3.44	5.57	-184.09	0.02	0.82
26	OwnSpecDens+ForestEdge+Pasture	4	376.38	3.50	5.74	-184.12	0.02	0.83
27	OwnSpecDens+CoastEdge+NDMI+NDVI+Pasture	6	376.45	3.56	5.93	-182.07	0.02	0.85
28	AllSpecDens+OwnSpecDens+CoastEdge+NDMI+Pasture	6	376.45	3.56	5.94	-182.07	0.02	0.86
29	AllSpecDens+OwnSpecDens+ForestEdge+NDMI+Pasture	6	376.49	3.60	6.06	-182.09	0.02	0.88

30	OwnSpecDens+CoastEdge+NDMI+Field+Pasture	6	376.49	3.60	6.06	-182.09	0.02	0.90
31	OwnSpecDens+ForestEdge+NDMI+NDVI+Pasture	6	376.55	3.66	6.24	-182.12	0.02	0.91
32	AllSpecDens+OwnSpecDens+Pasture	4	376.57	3.68	6.31	-184.21	0.02	0.93
33	OwnSpecDens+ForestEdge+NDMI+Field+Pasture	6	376.61	3.72	6.44	-182.15	0.01	0.94
34	OwnSpecDens+NDVI+Pasture	4	376.62	3.73	6.46	-184.24	0.01	0.96
35	AllSpecDens+OwnSpecDens+NDMI+NDVI+Pasture	6	376.62	3.73	6.46	-182.15	0.01	0.97
36	OwnSpecDens+Field+Pasture	4	376.67	3.78	6.62	-184.26	0.01	0.99
37	AllSpecDens+OwnSpecDens+NDMI+Field+Pasture	6	376.74	3.86	6.87	-182.22	0.01	1.00
**C) Collared flycatcher***								
*** Models were not analyzed due to the too small number of infected individuals (10)**								

3) *Trypanosoma*

Model	Terms	K	AICc	ΔAICc	Evidence ratio	Log-likelihood	ωAICc	Cumulative AICc
**A) Blue tit**								
1	AllSpecDens	2	64.63	0.00	1.00	-30.22	0.07	0.07
2	null	1	65.05	0.41	1.23	-31.49	0.06	0.13
3	AllSpecDens+NDVI	3	65.32	0.69	1.41	-29.47	0.05	0.18
4	NDVI	2	65.62	0.98	1.64	-30.72	0.04	0.23
5	AllSpecDens+NDVI+Pasture	4	66.05	1.42	2.03	-28.70	0.04	0.26
6	NDVI+Pasture	3	66.35	1.71	2.36	-29.98	0.03	0.29
7	CoastEdge+NDVI+Pasture	4	66.45	1.82	2.48	-28.90	0.03	0.32
8	AllSpecDens+CoastEdge	3	66.50	1.87	2.55	-30.06	0.03	0.35
9	AllSpecDens+Pasture	3	66.51	1.87	2.55	-30.06	0.03	0.38
10	CoastEdge	2	66.59	1.95	2.65	-31.20	0.03	0.40
11	AllSpecDens+CoastEdge+NDVI+Pasture	5	66.67	2.04	2.77	-27.84	0.03	0.43

12	AllSpecDens+OwnSpecDens	3	66.67	2.04	2.77	-30.15	0.03	0.46
13	AllSpecDens+Field	3	66.70	2.06	2.81	-30.16	0.03	0.48
14	AllSpecDens+NDMI	3	66.70	2.07	2.81	-30.16	0.03	0.51
15	AllSpecDens+ForestEdge	3	66.81	2.17	2.97	-30.21	0.02	0.53
16	Pasture	2	66.88	2.25	3.08	-31.35	0.02	0.55
17	OwnSpecDens	2	66.95	2.32	3.18	-31.38	0.02	0.58
18	ForestEdge	2	67.16	2.52	3.53	-31.49	0.02	0.60
19	Field	2	67.17	2.53	3.55	-31.49	0.02	0.62
20	NDMI	2	67.17	2.54	3.56	-31.49	0.02	0.64
21	CoastEdge+NDVI	3	67.38	2.74	3.94	-30.50	0.02	0.66
22	AllSpecDens+CoastEdge+NDVI	4	67.40	2.77	3.99	-29.38	0.02	0.67
23	AllSpecDens+OwnSpecDens+NDVI	4	67.52	2.88	4.23	-29.44	0.02	0.69
24	AllSpecDens+NDVI+Field	4	67.54	2.90	4.27	-29.45	0.02	0.71
25	AllSpecDens+ForestEdge+NDVI	4	67.55	2.91	4.29	-29.45	0.02	0.73
26	NDMI+NDVI	3	67.58	2.94	4.36	-30.60	0.02	0.74
27	AllSpecDens+NDMI+NDVI	4	67.58	2.94	4.36	-29.47	0.02	0.76
28	OwnSpecDens+NDVI	3	67.71	3.07	4.65	-30.66	0.02	0.77
29	NDVI+Field	3	67.80	3.17	4.87	-30.71	0.01	0.79
30	ForestEdge+NDVI	3	67.80	3.17	4.88	-30.71	0.01	0.80
31	AllSpecDens+CoastEdge+Pasture	4	67.87	3.24	5.05	-29.61	0.01	0.82
32	CoastEdge+Pasture	3	67.89	3.25	5.09	-30.75	0.01	0.83
33	NDMI+NDVI+Pasture	4	67.98	3.35	5.33	-29.67	0.01	0.85
34	ForestEdge+NDVI+Pasture	4	68.08	3.44	5.60	-29.72	0.01	0.86
35	AllSpecDens+ForestEdge+NDVI+Pasture	5	68.16	3.53	5.83	-28.59	0.01	0.87
36	AllSpecDens+NDMI+NDVI+Pasture	5	68.19	3.56	5.92	-28.60	0.01	0.88

37	AllSpecDens+OwnSpecDens+NDVI+Pasture	5	68.28	3.65	6.19	-28.65	0.01	0.89
38	OwnSpecDens+CoastEdge+NDVI+Pasture	5	68.34	3.70	6.37	-28.68	0.01	0.91
39	AllSpecDens+NDVI+Field+Pasture	5	68.36	3.72	6.44	-28.69	0.01	0.92
40	AllSpecDens+CoastEdge+NDMI	4	68.39	3.76	6.55	-29.87	0.01	0.93
41	ForestEdge+CoastEdge+NDVI+Pasture	5	68.42	3.79	6.65	-28.72	0.01	0.94
42	OwnSpecDens+NDVI+Pasture	4	68.44	3.80	6.70	-29.90	0.01	0.95
43	OwnSpecDens+CoastEdge	3	68.48	3.85	6.85	-31.05	0.01	0.96
44	AllSpecDens+OwnSpecDens+CoastEdge	4	68.54	3.90	7.04	-29.95	0.01	0.97
45	CoastEdge+NDVI+Field+Pasture	5	68.56	3.93	7.12	-28.79	0.01	0.98
46	AllSpecDens+OwnSpecDens+Pasture	4	68.58	3.95	7.20	-29.97	0.01	0.99
47	NDVI+Field+Pasture	4	68.60	3.97	7.26	-29.98	0.01	1.00
**B) Great tit**								
1	Pasture	2	178.49	0.00	1.00	-87.22	0.06	0.06
2	null	1	178.60	0.12	1.06	-88.29	0.05	0.11
3	CoastEdge	2	179.05	0.56	1.32	-87.50	0.04	0.15
4	Field+Pasture	3	179.59	1.10	1.74	-86.75	0.03	0.18
5	Field	2	179.70	1.22	1.84	-87.83	0.03	0.21
6	NDMI	2	180.01	1.53	2.15	-87.99	0.03	0.24
7	NDMI+Pasture	3	180.05	1.56	2.19	-86.98	0.03	0.27
8	CoastEdge+NDMI	3	180.10	1.61	2.24	-87.00	0.02	0.29
9	OwnSpecDens+Pasture	3	180.11	1.62	2.25	-87.01	0.02	0.31
10	ForestEdge	2	180.21	1.73	2.37	-88.09	0.02	0.34
11	CoastEdge+Pasture	3	180.23	1.74	2.39	-87.07	0.02	0.36
12	AllSpecDens+Pasture	3	180.34	1.85	2.53	-87.13	0.02	0.38
13	OwnSpecDens	2	180.50	2.01	2.73	-88.23	0.02	0.40

14	NDVI+Pasture	3	180.50	2.02	2.74	-87.21	0.02	0.42
15	ForestEdge+Pasture	3	180.52	2.04	2.77	-87.22	0.02	0.45
16	AllSpecDens	2	180.54	2.05	2.79	-88.25	0.02	0.47
17	NDVI	2	180.59	2.10	2.86	-88.27	0.02	0.48
18	CoastEdge+Field	3	180.60	2.12	2.88	-87.26	0.02	0.50
19	OwnSpecDens+CoastEdge	3	180.69	2.20	3.01	-87.30	0.02	0.52
20	NDMI+Field	3	180.85	2.36	3.26	-87.38	0.02	0.54
21	ForestEdge+Field	3	180.85	2.36	3.26	-87.38	0.02	0.56
22	NDMI+Field+Pasture	4	180.90	2.42	3.35	-86.38	0.02	0.57
23	AllSpecDens+CoastEdge	3	181.00	2.52	3.52	-87.46	0.02	0.59
24	ForestEdge+CoastEdge	3	181.02	2.53	3.54	-87.46	0.02	0.61
25	CoastEdge+NDVI	3	181.08	2.59	3.66	-87.50	0.02	0.62
26	OwnSpecDens+Field+Pasture	4	181.35	2.86	4.18	-86.60	0.01	0.63
27	NDVI+Field+Pasture	4	181.45	2.96	4.40	-86.65	0.01	0.65
28	CoastEdge+NDMI+Field	4	181.48	2.99	4.46	-86.66	0.01	0.66
29	AllSpecDens+Field+Pasture	4	181.51	3.02	4.53	-86.68	0.01	0.67
30	CoastEdge+NDMI+Pasture	4	181.56	3.07	4.65	-86.71	0.01	0.68
31	CoastEdge+Field+Pasture	4	181.58	3.09	4.69	-86.71	0.01	0.70
32	ForestEdge+Field+Pasture	4	181.62	3.13	4.79	-86.73	0.01	0.71
33	ForestEdge+NDMI	3	181.67	3.19	4.92	-87.79	0.01	0.72
34	OwnSpecDens+NDMI+Pasture	4	181.68	3.20	4.94	-86.77	0.01	0.73
35	OwnSpecDens+Field	3	181.69	3.20	4.96	-87.80	0.01	0.74
36	OwnSpecDens+CoastEdge+NDMI	4	181.69	3.20	4.96	-86.77	0.01	0.75
37	AllSpecDens+Field	3	181.70	3.21	4.98	-87.80	0.01	0.76
38	NDVI+Field	3	181.74	3.26	5.10	-87.83	0.01	0.77

39	OwnSpecDens+CoastEdge+Pasture	4	181.77	3.29	5.18	-86.81	0.01	0.79
40	OwnSpecDens+NDMI	3	181.90	3.41	5.51	-87.90	0.01	0.80
41	AllSpecDens+NDMI+Pasture	4	181.96	3.48	5.69	-86.91	0.01	0.81
42	AllSpecDens+NDMI	3	181.99	3.51	5.77	-87.95	0.01	0.82
43	ForestEdge+NDMI+Field	4	182.00	3.51	5.79	-86.92	0.01	0.82
44	NDMI+NDVI	3	182.02	3.53	5.85	-87.97	0.01	0.83
45	OwnSpecDens+ForestEdge	3	182.02	3.54	5.86	-87.97	0.01	0.84
46	NDMI+NDVI+Pasture	4	182.08	3.59	6.02	-86.96	0.01	0.85
47	ForestEdge+NDMI+Pasture	4	182.11	3.62	6.11	-86.98	0.01	0.86
48	ForestEdge+CoastEdge+NDMI	4	182.11	3.62	6.12	-86.98	0.01	0.87
49	AllSpecDens+CoastEdge+NDMI	4	182.12	3.63	6.14	-86.98	0.01	0.88
50	AllSpecDens+ForestEdge	3	182.12	3.63	6.15	-88.01	0.01	0.89
51	AllSpecDens+CoastEdge+Pasture	4	182.12	3.64	6.16	-86.99	0.01	0.90
52	CoastEdge+NDMI+NDVI	4	182.14	3.65	6.21	-86.99	0.01	0.91
53	AllSpecDens+OwnSpecDens+Pasture	4	182.15	3.66	6.24	-87.00	0.01	0.92
54	OwnSpecDens+NDVI+Pasture	4	182.16	3.67	6.27	-87.00	0.01	0.93
55	OwnSpecDens+ForestEdge+Pasture	4	182.17	3.68	6.31	-87.01	0.01	0.93
56	ForestEdge+NDVI	3	182.20	3.71	6.40	-88.06	0.01	0.94
57	CoastEdge+NDVI+Pasture	4	182.24	3.75	6.52	-87.04	0.01	0.95
58	ForestEdge+CoastEdge+Pasture	4	182.27	3.78	6.62	-87.06	0.01	0.96
59	ForestEdge+CoastEdge+Field	4	182.33	3.84	6.83	-87.09	0.01	0.97
60	AllSpecDens+NDVI+Pasture	4	182.36	3.87	6.93	-87.10	0.01	0.98
61	AllSpecDens+ForestEdge+Pasture	4	182.39	3.91	7.06	-87.12	0.01	0.98
62	OwnSpecDens+CoastEdge+Field	4	182.39	3.91	7.06	-87.12	0.01	0.99
63	OwnSpecDens+NDVI	3	182.48	3.99	7.36	-88.19	0.01	1.00

C) Collared flycatcher

1	CoastEdge	2	299.53	0.00	1.00	-147.74	0.11	0.11
2	OwnSpecDens+CoastEdge	3	300.83	1.30	1.91	-147.37	0.06	0.17
3	CoastEdge+NDMI	3	301.08	1.55	2.17	-147.49	0.05	0.22
4	ForestEdge+CoastEdge	3	301.08	1.55	2.17	-147.49	0.05	0.27
5	CoastEdge+Field	3	301.08	1.55	2.18	-147.50	0.05	0.32
6	CoastEdge+NDVI	3	301.19	1.66	2.29	-147.55	0.05	0.37
7	AllSpecDens+CoastEdge	3	301.45	1.92	2.61	-147.68	0.04	0.42
8	CoastEdge+Pasture	3	301.56	2.03	2.76	-147.73	0.04	0.46
9	AllSpecDens+OwnSpecDens+CoastEdge	4	301.80	2.27	3.11	-146.82	0.04	0.49
10	OwnSpecDens+ForestEdge+CoastEdge	4	302.22	2.69	3.83	-147.03	0.03	0.52
11	OwnSpecDens+CoastEdge+Field	4	302.41	2.88	4.23	-147.13	0.03	0.55
12	CoastEdge+NDMI+NDVI	4	302.45	2.92	4.31	-147.15	0.03	0.57
13	OwnSpecDens+CoastEdge+NDMI	4	302.48	2.95	4.37	-147.16	0.03	0.60
14	ForestEdge+CoastEdge+Field	4	302.51	2.98	4.45	-147.18	0.03	0.62
15	ForestEdge+CoastEdge+NDMI	4	302.57	3.04	4.57	-147.21	0.02	0.65
16	CoastEdge+NDMI+Field	4	302.66	3.13	4.78	-147.25	0.02	0.67
17	OwnSpecDens+CoastEdge+NDVI	4	302.67	3.14	4.81	-147.26	0.02	0.70
18	ForestEdge+CoastEdge+NDVI	4	302.78	3.25	5.07	-147.31	0.02	0.72
19	CoastEdge+NDVI+Field	4	302.83	3.30	5.20	-147.34	0.02	0.74
20	null	1	302.85	3.32	5.26	-150.42	0.02	0.76
21	OwnSpecDens+CoastEdge+Pasture	4	302.88	3.35	5.33	-147.36	0.02	0.78
22	ForestEdge+CoastEdge+Pasture	4	302.99	3.46	5.63	-147.42	0.02	0.80
23	AllSpecDens+CoastEdge+NDMI	4	303.03	3.50	5.75	-147.44	0.02	0.82
24	AllSpecDens+CoastEdge+Field	4	303.04	3.51	5.78	-147.44	0.02	0.84

25	AllSpecDens+ForestEdge+CoastEdge	4	303.06	3.53	5.84	-147.45	0.02	0.86
26	AllSpecDens+CoastEdge+NDVI	4	303.13	3.60	6.05	-147.49	0.02	0.88
27	CoastEdge+NDMI+Pasture	4	303.14	3.61	6.07	-147.49	0.02	0.90
28	CoastEdge+Field+Pasture	4	303.14	3.61	6.07	-147.49	0.02	0.91
29	CoastEdge+NDVI+Pasture	4	303.14	3.61	6.08	-147.49	0.02	0.93
30	Pasture	2	303.15	3.62	6.12	-149.55	0.02	0.95
31	AllSpecDens+OwnSpecDens+ForestEdge+CoastEdge	5	303.23	3.70	6.36	-146.50	0.02	0.97
32	AllSpecDens+OwnSpecDens+CoastEdge+Field	5	303.48	3.95	7.20	-146.62	0.02	0.98
33	AllSpecDens+CoastEdge+Pasture	4	303.48	3.95	7.20	-147.66	0.02	1.00

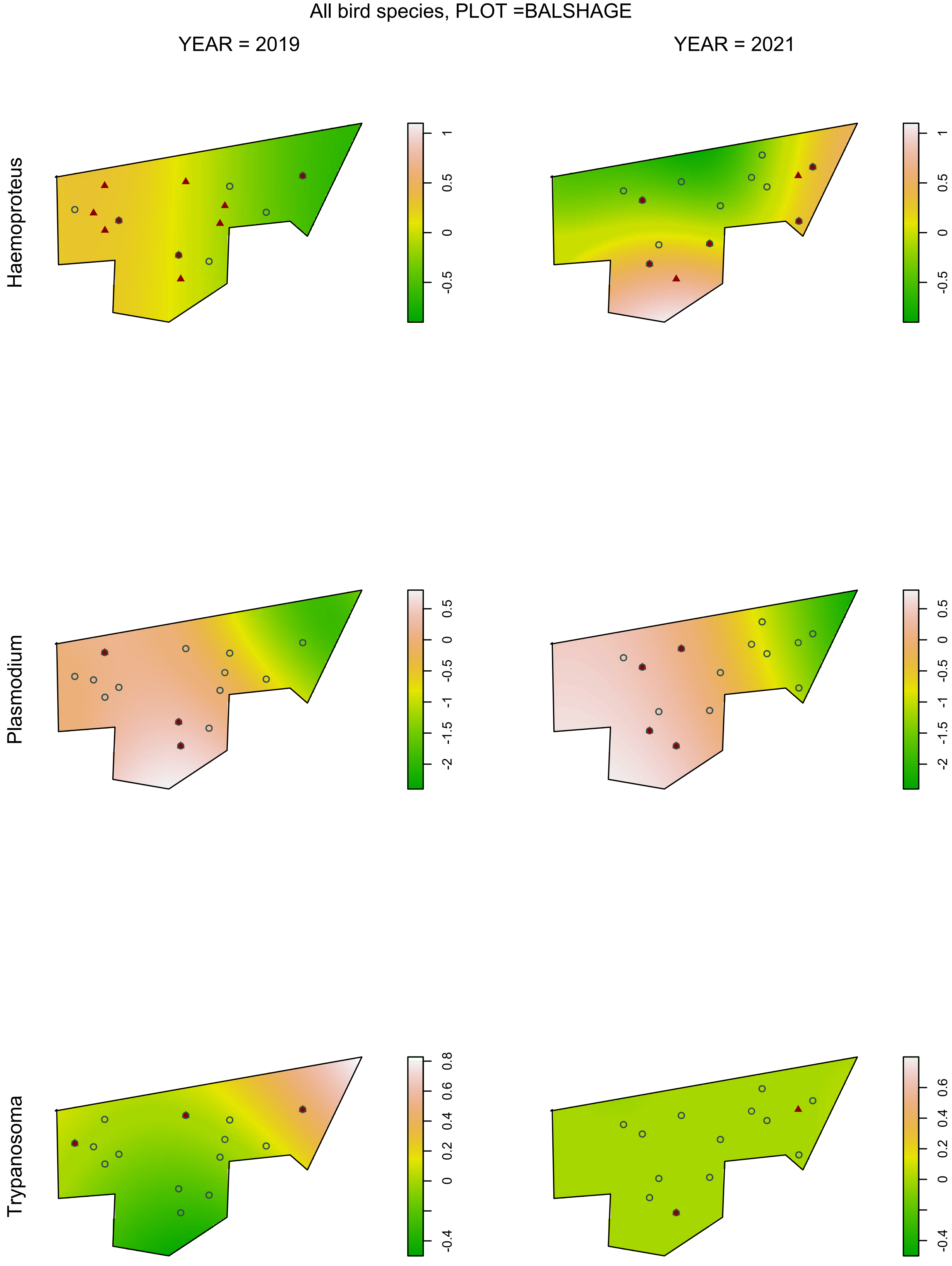

All bird species, PLOT =BALSHAGE
YEAR = 2019
YEAR = 2021
Haemoproteus
Plasmodium
Trypanosoma
1
0.5
0
-0.5
-2
-1.5
-1
0.8
0.6
0.4
0.2
-0.4

All bird species, PLOT =DOMERARVE

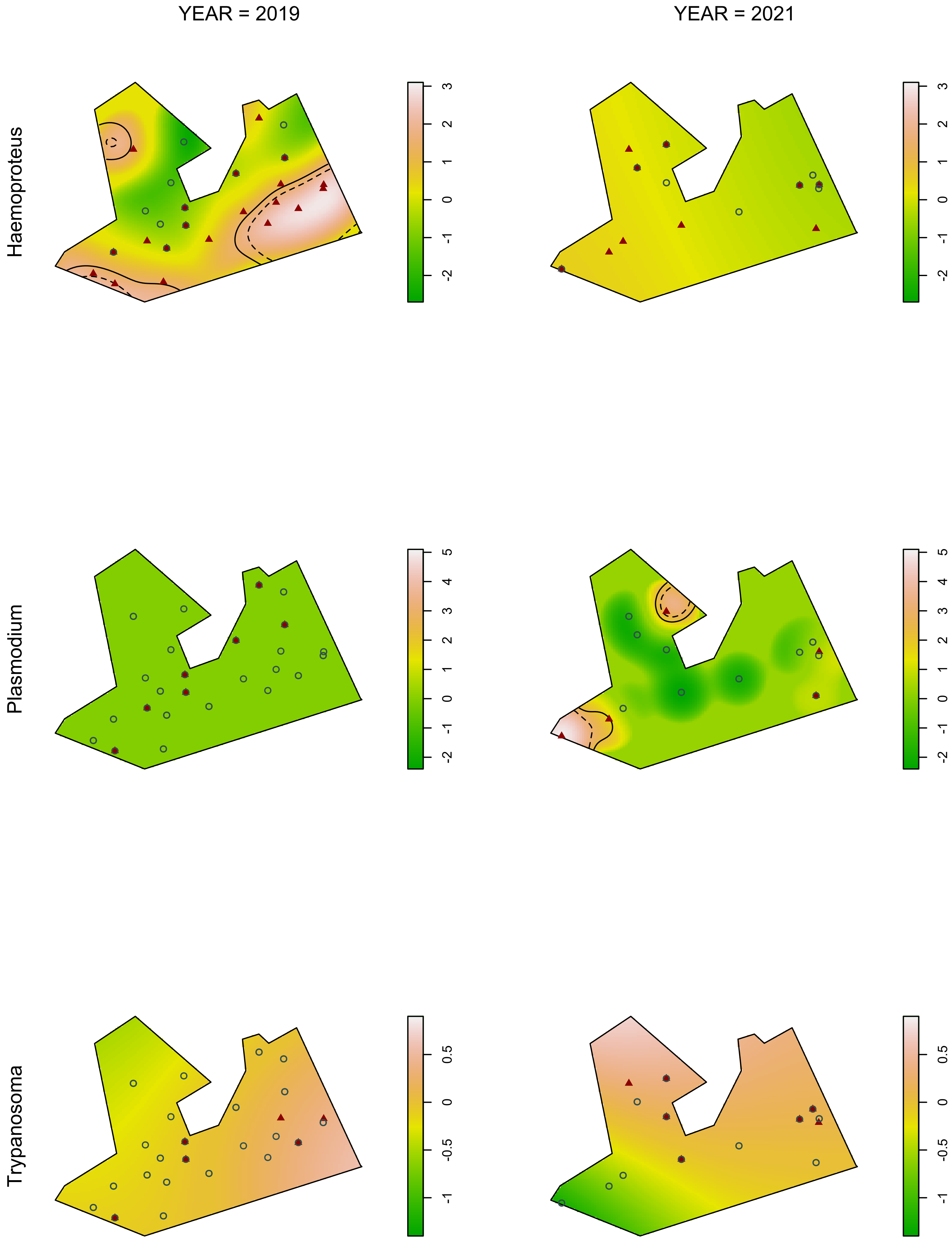

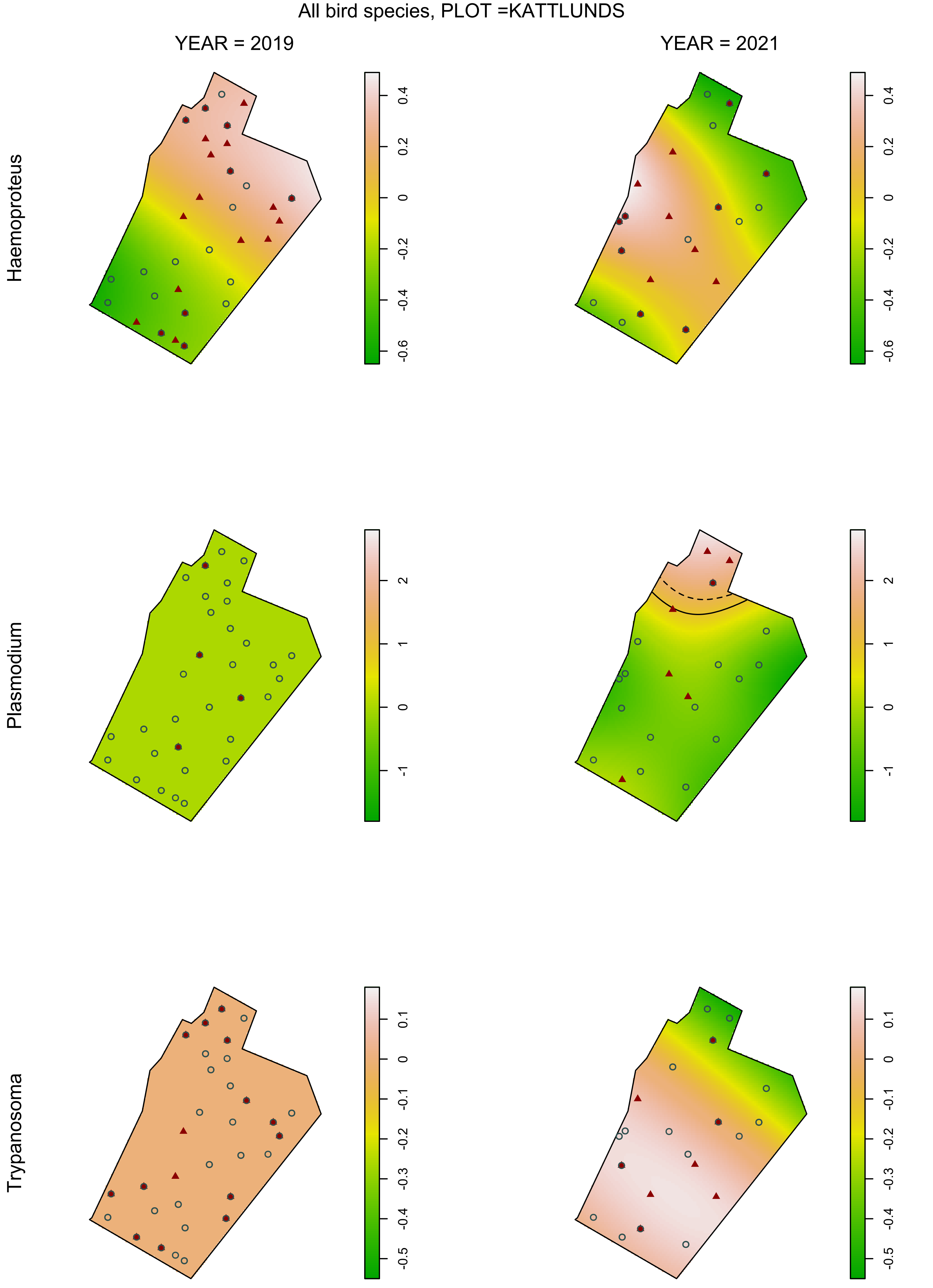
All bird species, PLOT =KATTLUNDS
YEAR = 2019
YEAR = 2021
Haemoproteus
Plasmodium
Trypanosoma
0.4
0.2
0
-0.2
-0.4
-0.6
2
1
0
-1
0.1
0
-0.1
-0.2
-0.3
-0.4
-0.5

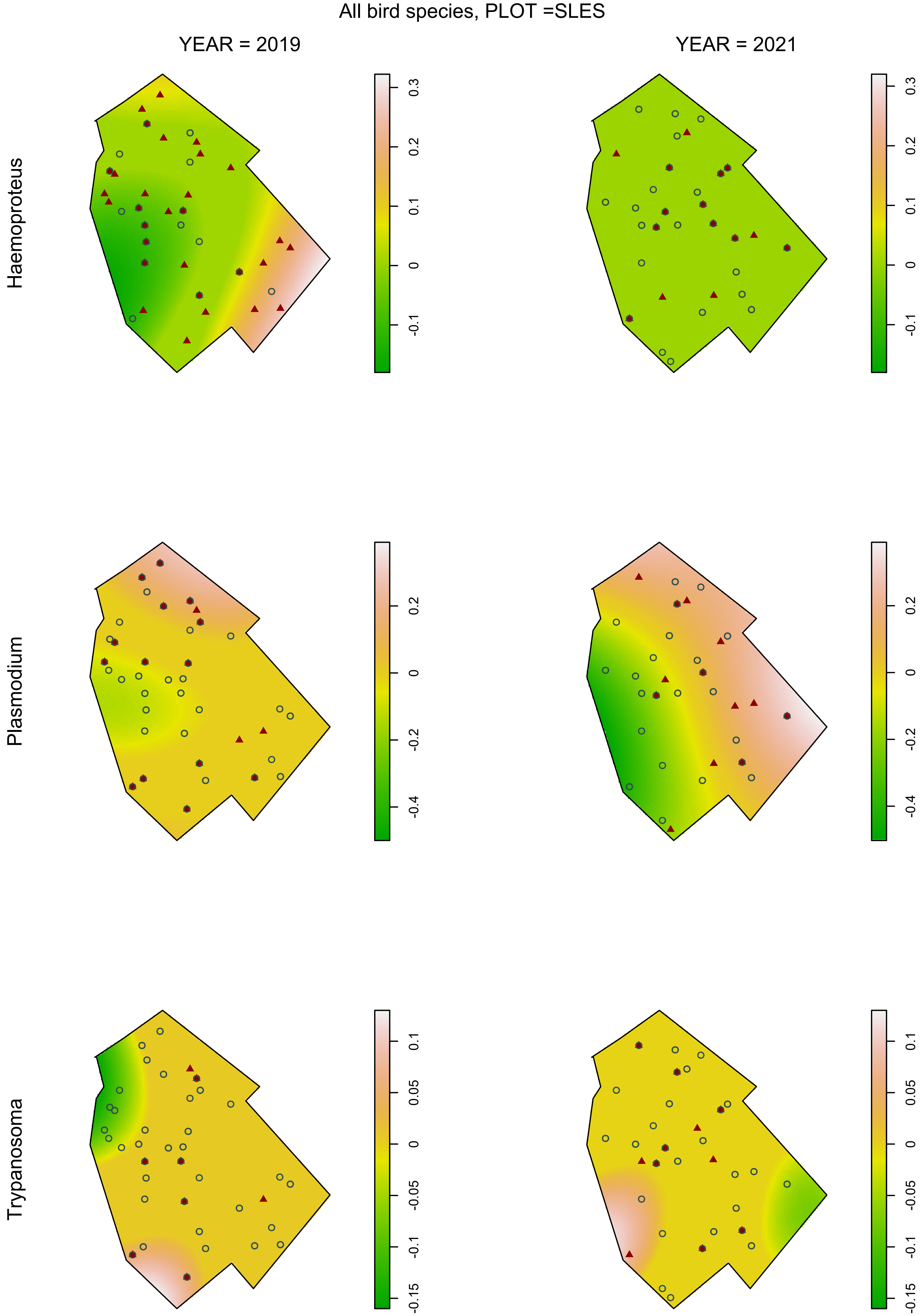
All bird species, PLOT =SLES
YEAR = 2019
YEAR = 2021
Haemoproteus
Plasmodium
Trypanosoma
0.3
0.2
0.1
0
-0.1
0.2
0
-0.2
-0.4
0.1
0.05
0
-0.05
-0.1
-0.15

Figure S2. Kernel-smoothed maps showing log-relative risk of infection with *Haemoproteus*, *Plasmodium* and *Trypanosoma* in each year of the study, across the four study plot (except the largest - Rums shown in the main text) for all bird species. Value ranges (depicted by colours) have been scaled to the maximum and minimum values observed across both years separately for each parasite genus. Contour lines depict areas of significantly elevated relative risk (solid line for $p < 0.05$; dashed line for $p < 0.01$). The locations of infected individuals are shown by red filled triangles; uninfected ones by open circles.
